# PAD2-Mediated Citrullination Contributes to Efficient Oligodendrocyte Differentiation and Myelination

**DOI:** 10.1016/j.celrep.2019.03.108

**Published:** 2019-04-23

**Authors:** Ana Mendanha Falcão, Mandy Meijer, Antonella Scaglione, Puneet Rinwa, Eneritz Agirre, Jialiang Liang, Sara C. Larsen, Abeer Heskol, Rebecca Frawley, Michael Klingener, Manuel Varas-Godoy, Alexandre A.S.F. Raposo, Patrik Ernfors, Diogo S. Castro, Michael L. Nielsen, Patrizia Casaccia, Gonçalo Castelo-Branco

**Affiliations:** 1 Laboratory of Molecular Neurobiology, Department of Medical Biochemistry and Biophysics, Karolinska Institutet, 17177 Stockholm, Sweden; 2 Neuroscience Initiative at the Advanced Science Research Center of the Graduate Center of the City University of New York, New York, NY, USA; 3 Department of Proteomics, the Novo Nordisk Foundation Center for Protein Research, Faculty of Heath Sciences, University of Copenhagen, Blegdamsvej 3B, 2200 Copenhagen, Denmark; 4 Cancer Cell Biology Lab, Centro de Biología Celular y Biomedicina (CEBICEM), Facultad de Medicina y Ciencia, Universidad San Sebastián, Santiago 7510157, Chile; 5 Instituto Gulbenkian de Ciência, 2780-156 Oeiras, Portugal; 6 Ming Wai Lau Centre for Reparative Medicine, Stockholm Node, Karolinska Institutet, 171 77 Stockholm, Sweden; 7 Graduate School of Biomedical Sciences, Icahn School of Medicine at Mount Sinai, New York, NY, 10029

**Keywords:** oligodendrocytes, myelin, Padi2, PAD2, citrullination, deamination, chromatin accessibility, multiple sclerosis, proteomics, SILAC

## Abstract

Citrullination, the deimination of peptidylarginine residues into peptidylcitrulline, has been implicated in the etiology of several diseases. In multiple sclerosis, citrullination is thought to be a major driver of pathology through hypercitrullination and destabilization of myelin. As such, inhibition of citrullination has been suggested as a therapeutic strategy for MS. Here, in contrast, we show that citrullination by peptidylarginine deiminase 2 (PAD2) contributes to normal oligodendrocyte differentiation, myelination, and motor function. We identify several targets for PAD2, including myelin and chromatin-related proteins, implicating PAD2 in epigenomic regulation. Accordingly, we observe that PAD2 inhibition and its knockdown affect chromatin accessibility and prevent the upregulation of oligodendrocyte differentiation genes. Moreover, mice lacking PAD2 display motor dysfunction and a decreased number of myelinated axons in the corpus callosum. We conclude that citrullination contributes to proper oligodendrocyte lineage progression and myelination.

## Highlights

• PAD2 is increased upon OL differentiation
• OL differentiation is facilitated by PAD2-mediated chromatin remodeling in myelin genes
• PAD2 contributes to efficient myelination and motor and cognitive functions
• Nuclear and myelin proteins interact and are citrullinated by PAD2

## Graphical Abstract

**Figure:**
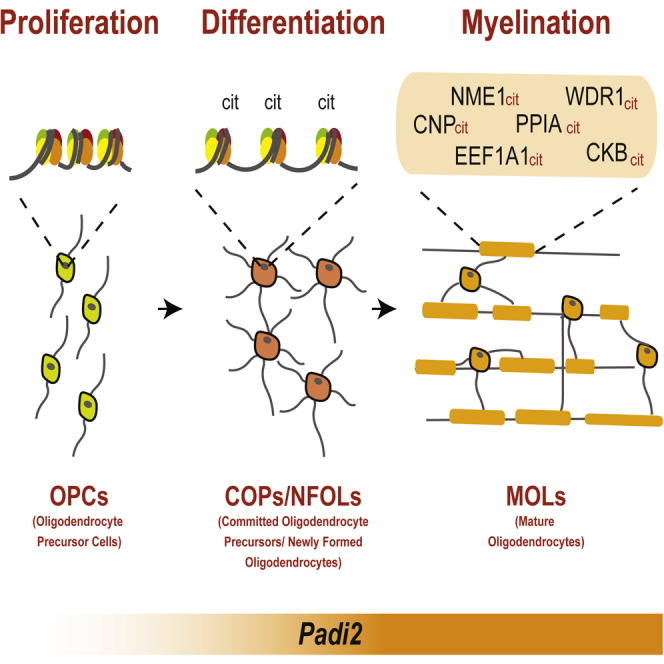

## Teaser

Falcão et al. demonstrate that PAD2-mediated citrullination, the conversion of peptidylarginine into peptidylcitrulline, has two roles in oligodendrocyte cells. In the nucleus, PAD2 acts as a chromatin modifier contributing to efficient oligodendrocyte precursor cell differentiation, while in the cytoplasm PAD2 is required for proper oligodendrocyte myelination.

## Introduction

The conversion of a peptidylarginine into a peptidylcitrulline can introduce profound changes in the structure and function of the modified protein. This posttranslational modification, also known as protein citrullination or deimination, is calcium dependent and is mediated by the family of enzymes called peptidylarginine deiminases (*Padi*s or PADs) (Wang and Wang, 2013). Five PAD isozymes (PAD1, PAD2, PAD3, PAD4, and PAD6) have been identified in mammals, and their expression patterns and function are tissue specific (Vossenaar et al., 2003). Citrullination has been recently shown to play key roles in multiple cellular processes, such as inflammatory immune responses (Neeli et al., 2008), apoptosis (Liu et al., 2006), and regulation of pluripotency (Christophorou et al., 2014). In the central nervous system (CNS), PAD2 is the most prevalent expressed PAD and occurs preferentially in oligodendrocytes (OLs) and other glial cells (Zeisel et al., 2015). OLs arise at postnatal stages from the differentiation of OL precursor cells (OPCs), and their most prominent function is the formation of the myelin sheath, an electrical insulating layer for the axons, essential for proper neuronal communication and thus CNS function (Bergles and Richardson, 2015).

PAD2 has been reported to heavily citrullinate myelin binding protein (MBP), a fundamental myelin component. The ratio of citrullinated MBP (MBPcit) to total MBP (MBPtotal) is high in the first 4 years of life and similarly in multiple sclerosis (MS), a disease in which demyelination occurs (Moscarello et al., 1994). Upon citrullination, MBP arginine residues lack positive charges and thereby have reduced interaction with negatively charged lipids. Consequently, MBPcit does not form compact sheaths, and the myelin remains immature or becomes unstable (Beniac et al., 2000). In accordance, transgenic mice overexpressing PAD2 in OL cells display myelin loss (Musse et al., 2008). In light of this, PADs, in particular PAD2, have been thought to have a detrimental effect in MS, and thus alleviation of symptoms can be achieved by inhibiting their actions (Caprariello et al., 2018). Indeed, treatment of an MS mouse model of experimental autoimmune encephalomyelitis (EAE) with 2-chloroacetamidine (2-CA), a PAD2 and PAD4 inhibitor, leads to disease attenuation (Moscarello et al., 2013). The reduction of disease progression by 2-CA treatment was not specifically assigned to the lack of PAD activity in OL lineage cells alone but to inhibition of all PAD-expressing cells, including immune cells that play an essential role in disease development.

Interestingly, however, no changes in disease progression were observed upon EAE induction in PAD2 KO mice (Raijmakers et al., 2006). This could point to a possible compensation of PAD2 functions by other PADs or to additional functions of PAD2 in EAE disease progression. Although citrullination of myelin might play an important role in MS, other proteins, such as histone H3, have been found to be citrullinated in MS (Mastronardi et al., 2006). Thus, the role of PAD2-mediated citrullination in MS might not be confined to myelin. Furthermore, although the effects of *Padi2* overexpression in mature OLs have been characterized, its absence in OL lineage cells has not been further investigated, nor its physiological function in OL lineage cells and its significance for myelin integrity maintenance.

## Results

### *Padi2* Expression Is Increased upon OL Differentiation

**Figure 1:**
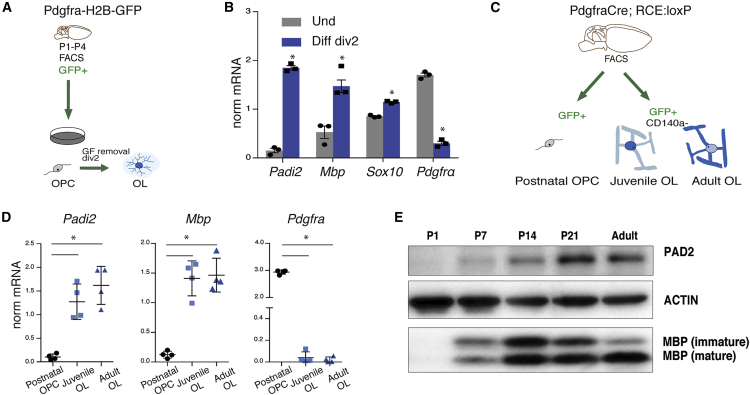
Padi2 Expression Is Substantially Increased upon OL Differentiation (A) Schematic representation of the methodology used for OPC *in vitro* cultures. P1–P4 GFP^+^ OPCs are dissociated from brains of the transgenic mice line Pdgfra-H2B-GFP and FACS-sorted to plates to expand in the presence of growth factors (GFs). GFs are removed to induce differentiation for 2 days. (B) Comparative gene expression analysis of OPCs and 2 day differentiated OLs. Means ± SEM are shown, n = 3; ^∗^p < 0.05, two-tailed t test. (C) Schematic representation of the methodology used to specifically isolate OPCs and juvenile and adult OLs from the postnatal (P1–P4), juvenile (P21), and adult (P60) brains of the transgenic mice PdgfraCre;RCE:loxP (R26R CAG-boosted EGFP); GFP^+^ cells were depleted of the OPC marker CD140a to specifically isolate OLs. (D) Comparative gene expression analysis of OPCs and juvenile and adult OLs. Means ± SEM are shown, n = 4; ^∗^p < 0.05, one-way non-parametric ANOVA. (E) Western blot for PADI2 and MBP on the spinal cords of P1, P7, P14, P21, and adult wild-type mice. ACTIN signal is an internal loading control. See also Figure S1.

By analyzing our single-cell RNA sequencing (RNA-seq) dataset of the OL lineage in the adult and juvenile mouse brain (Marques et al., 2016), we identified *Padi2* as the predominant *Padi* expressed in OLs (Figure S1A). Interestingly, *Padi2* expression is found in OPCs, increases in committed OL precursors (COPs) and newly formed OLs (NFOLs), and peaks at more mature stages (Figure S1A). Surprisingly, we did not observe expression of *Padi4*, which has been previously suggested to be present in myelin (Mastronardi et al., 2006, Wood et al., 2008). To further investigate the pattern of expression of *Padi2* during early OL lineage progression, we cultured OPCs isolated from postnatal day (P) 1 to P4 brains of the transgenic mouse line *Pdgfra*-histone 2b (H2B)-GFP (Klinghoffer et al., 2002) in which nuclear GFP expression is under the control of the endogenous *Pdgfra* promoter locus. GFP^+^ OPCs were collected with fluorescence-activated cell sorting (FACS) to plates and expanded in media containing the growth factors (GFs) basic fibroblast growth factor (bFGF) and platelet-derived growth factor (PDGF)-AA and differentiated into OLs by removing the GFs for 2 days (Figure 1A). Gene expression of the differentiation markers *Mbp* and *Sox10* was upregulated, and the progenitor marker *Pdgfra* was reduced upon GF removal (Figure 1B). In agreement with the single-cell RNA-seq data, *Padi2* was expressed in OPCs, and it was greatly enhanced upon differentiation (Figure 1B; Figure S1B, for the mouse oligodendroglia cell line Oli-neu; Jung et al., 1995). To investigate *Padi2* expression in the OL lineage *in vivo*, we isolated OPCs and OLs from the brain of the transgenic mouse line *Pdgfra*-Cre-loxP-GFP (Kang et al., 2010), in which all OL lineage cells express the GFP reporter (Figure 1C). GFP^+^ OPCs were collected with FACS from P1–P4 pups (all GFP^+^ cells are OPCs because OLs are not formed yet), and GFP^+^ CD140a^−^ OLs were collected with FACS from juvenile (P21) and adult (P60) mice (at this stage, GFP^+^ cells comprise all OL progeny, including CD140a^+^ OPCs). As a confirmation of the sorted populations, we assessed the expression of *Pdgfra* and *Mbp* as markers for OPCs and differentiated OLs, respectively (Figure 1D). *Padi2* mRNA was substantially enriched in OLs from both juvenile and adult brains compared with postnatal OPCs (Figure 1D). At the protein level, and in agreement with our gene expression data, we observed a continuous increase in PAD2 protein from P1 to adult in the spinal cord of wild-type mice, concomitant with the increase in the OL marker MBP (Figure 1E). Thus, PAD2 is rapidly upregulated upon OPC differentiation, suggesting a role of this citrullinating enzyme at this stage of OL lineage progression.

### Reduction of PAD2 Activity Hinders OL Differentiation

**Figure 2:**
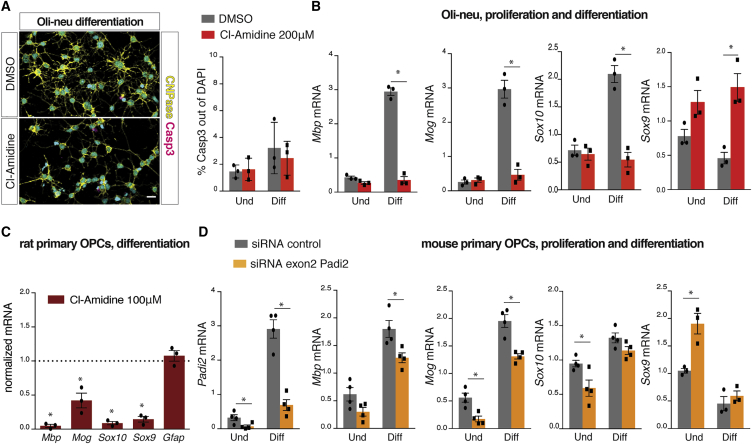
PAD Inhibition and Padi2 Knockdown Hinders OPC Differentiation (A) Immunocytochemistry for CNPase and cleaved Casp3 on 2 day differentiated Oli-neu upon 2 days treatment with DMSO or Cl-amidine 200 μM and quantification of the percentage of cleaved Casp3^+^ cells out of total DAPI in all conditions. Scale bar, 20 μm. (B) Comparative gene expression analysis of proliferating and undifferentiated (Und) Oli-neu cells and 2 day differentiated Oli-neu cells (Diff) treated with DMSO or Cl-amidine 200 μM for 2 days. Means ± SEM are shown, n = 3; ^∗^p < 0.05, two-tailed t test. (C) Gene expression analysis on proliferating rat primary OPCs treated with Cl-amidine 100 μM; dashed line represents control levels, normalized to 1. Means ± SEM are shown, n = 3; ^∗^p < 0.05, one-sample t test. (D) Comparative gene expression analysis on mouse primary undifferentiated (Und) and 2 day differentiated OLs (Diff) transfected with either scrambled siRNA or Padi2 siRNA targeting exon 2. Means ± SEM are shown, n = 4; ^∗^p < 0.05, two-tailed t test; error bars represent SEM. See also Figure S1.

In order to investigate whether the increased expression of *Padi2* upon OL differentiation has functional significance, we inhibited PAD activity in mouse Oli-neu cells and rat primary OPCs using Cl-amidine, a pan-inhibitor of PAD enzymes. The concentration used of PAD inhibitor (200 μM) has been used in other studies (Christophorou et al., 2014, Xiao et al., 2017) and had no effect on cell death in Oli-neu cells, as assessed using cleaved CASP3 immunostaining (Figure 2A). Strikingly, Oli-neu cell differentiation was compromised in the presence of this inhibitor, as observed by the reduction of cell branching typical of differentiating OLs shown with CNPase immunostaining (Figure 2A). In accordance, gene expression analysis on undifferentiated and differentiated Oli-neu cells treated with Cl-amidine revealed a striking impairment of OPC differentiation. The increase of *Mbp*, *Sox10*, and *Mog* mRNA levels, crucial for proper OPC differentiation, were no longer observed after Cl-amidine during differentiation (Figure 2B). Interestingly, the expression of the transcription factor (TF) *Sox9*, required for OL specification (Stolt et al., 2003) and downregulated upon OPC differentiation (Marques et al., 2016), was increased upon PAD inhibition (Figure 2B). Because *Sox9* is highly expressed in astrocytes, we further investigated if the upregulation of *Sox9* drove OPCs into astrocytes. We did not observe any upregulation of the astrocytic marker *Gfap* upon PAD inhibition, ruling out a possible differentiation of OPCs into astrocytes instead of OLs (Figure S1D). Similar results on impairment of OPC differentiation were obtained by treating Oli-neu with another PAD inhibitor, 2-CA (Figure S1C). Importantly, these effects were also observed in rat OPC primary cultures, in which under differentiating conditions, treatment with 50 or 100 μM Cl-amidine impaired differentiation, as observed by the lower mRNA expression levels of the differentiation markers *Mbp*, *Sox10*, and *Mog* (Figure 2C). In contrast to Oli-neu, *Sox9* was reduced upon PAD2 inhibition in rat OPC primary cultures (Figure 2C).

To investigate whether the effect of PAD inhibitors was due to PAD2 inhibition, we specifically decreased *Padi2* mRNA levels in mouse primary OPCs by transfecting cells with small interfering RNA (siRNA) against *Padi2* (targeting exon 2). Similar to PAD inhibition, the reduction of *Padi2* expression led to decreases in the mRNA levels of *Sox10* and *Mog* and an increase in *Sox9* without altering *Gfap* mRNA levels (Figures 2D and S1F). *Mog*, *Mbp*, and *Sox10* mRNA levels were also reduced in *Padi2* knockdown conditions after 2 days of differentiation (Figure 2D). Likewise, *Padi2* knockdown in Oli-neu cells mimicked the effects of PAD inhibition (Figure S1E). The results with inhibitors in Oli-neu are stronger than in primary OPCs, most likely because the inhibitors act in a quicker manner and irreversibly inhibit all PAD2 present in Oli-neu cells, whereas *Padi2* knockdown is slower and not as effective in reducing PAD2 activity. Thus, our findings indicate that PAD2 is dramatically increased at the onset of OL differentiation, when it contributes to the transition from an OPC state to an NFOL state.

### OLs Are Transiently Reduced in the Spinal Cord of Juvenile Mice upon Conditional *Padi2* Knockout in the OL Lineage

**Figure 3:**
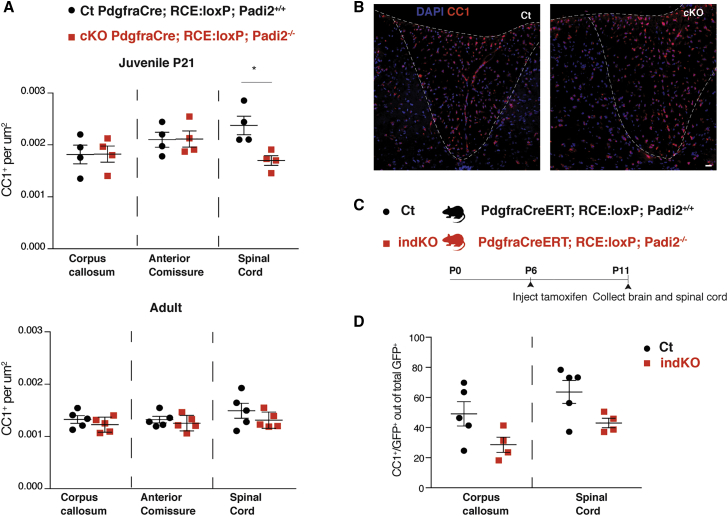
Padi2 Knockout Mice Display a Transient Decrease in OPC Differentiation in the Spinal Cord (A) CC1^+^ cells are presented as number of positive cells per area in the corpus callosum, anterior commissure, and spinal cord of PdgfraCre;RCE:loxP;Padi2^+/+^ and PdgfraCre;RCE:loxP;Padi2^−/−^ juvenile (P21) and adult mice. Means ± SEM are shown, n = 4 (P21) and n = 5 (adult); ^∗^p < 0.05, two-tailed t test. (B) Representative images of the spinal cord dorsal funiculus of PdgfraCre;RCE:loxP;Padi2^+/+^ and PdgfraCre;RCE:loxP;Padi2^−/−^ mice stained with CC1 antibody and DAPI. Scale bar, 20 μm. (C) Schematic representation of the strategy used to deplete Padi2 immediately before the peak of OPC differentiation: PdgfraCreERT;RCE:loxP;Padi2^+/+^ and PdgfraCreERT;RCE:loxP;Padi2^−/−^ mice were injected with tamoxifen at postnatal day 6 (P6) and sacrificed at P11, and brains and spinal cords were collected. (D) The number of CC1 and GFP double-positive cells was estimated out of total GFP^+^ cells in both corpus callosum and spinal cord of PdgfraCreERT;RCE:loxP;Padi2^+/+^ and PdgfraCreERT;RCE:loxP;Padi2^−/−^ mice. Means ± SEM are shown, n = 5 (control [Ct]) and n = 4 (indKO); ^∗^p < 0.05, two-tailed t test. See also Figure S2.

To investigate if the observed effects of PAD2 have an impact on OL differentiation and myelin maintenance *in vivo*, we generated a *Padi2* conditional knockout (cKO) mouse line, *Pdgfra*Cre;RCE:loxP;*Padi2*^−/−^, in which *Padi2* is not present in any cells derived from *Pdgfra*^+^ cells, which include the whole OL lineage. We estimated the number of CC1^+^ cells per area at P21, in the corpus callosum, anterior commissure, and dorsal funiculus of the spinal cord. In accordance with our results in Oli-neu and primary OPC cultures (Figure 2), we observed a reduction in the number of CC1^+^ OLs in the spinal cord of PAD2 cKO mice, compared with littermate controls (Figures 3A and 3B). Nevertheless, we did not observe such an effect in the brain (corpus callosum and anterior commissure) (Figure 3A). Accordingly, we observed that *Pdgfra*, *Mbp*, and *Mog* mRNA were unaltered in FACS cKO GFP^+^ cells from P21 brains (Figure S2C), indicating that the *Padi2* cKO does not affect OL cell number or transcription in the brain but rather has a more prominent effect in OPC differentiation in posterior regions of the CNS. To further address if the effect in the spinal cord is sustained in adulthood, we estimated CC1^+^ cell densities in 4-month-old mice. We did not observe any difference in the three regions analyzed (Figure 3A), suggesting that there is a recovery in the number of generated CC1^+^ cells in adult stages in the spinal cord.

We also generated an inducible *Padi2* cKO (indKO) mouse line, *Pdgfra*CreERT;RCE:loxP;*Padi2*^−/−^, for which *Padi2* could be ablated from OPCs by tamoxifen treatment at early postnatal stages (Figure 3C). Because we observed that PAD2 mainly modulates the transition between OPCs and NFOLs *in vitro* (Figure 2), we depleted *Padi2* specifically at the onset of OPC differentiation by injecting mice with tamoxifen at P6 and collecting the brains and spinal cord at P11 (Figure 3C). We observed a slight effect of the number of OLs formed upon tamoxifen-induced OPC *Padi2* depletion, which nevertheless was not statistically significant (p = 0.0828 for corpus callosum and p = 0.0567 for spinal cord; Figure 3D). Thus, our results with *Padi2* constitutive ablation in OPC suggest that PAD2 facilitates the generation of OLs *in vivo* in the spinal cord.

### *Padi2* KO Mice Display Impaired Motor and Cognitive Functions and a Decrease in the Number of Myelinated Axons

**Figure 4:**
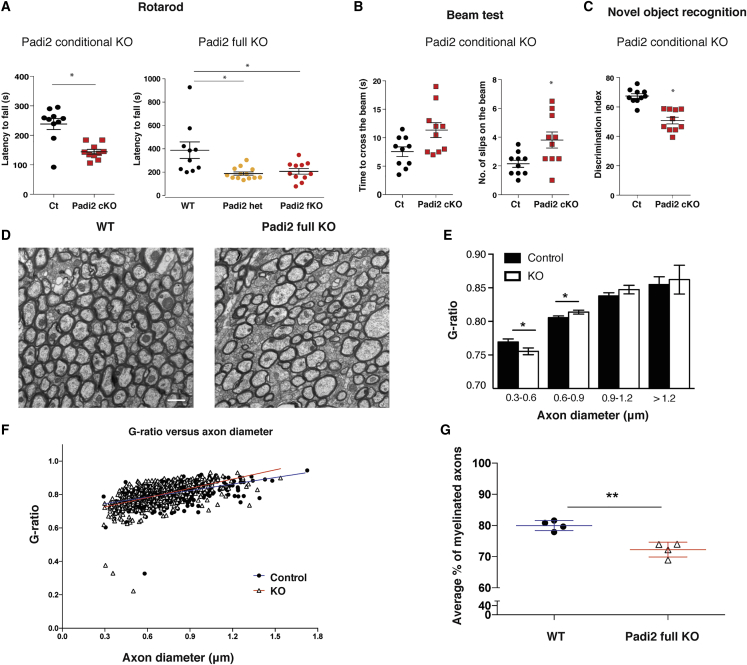
Padi2 Conditional and Full Knockout Mice Exhibit Impaired Motor and Cognitive Functions and Decreased Myelinated Axons (A–C) Behavior tests for motor (rotarod [A] and beam test [B]) and cognitive (novel object recognition [C]) performance in controls (PdgfraCre;RCE:loxP;Padi2^+/+^ and wild-type [WT]), Padi2 heterozygous (Padi2 het), and Padi2 full knockout (fKO) and conditional KO (Padi2 cKO; PdgfraCre;RCE:loxP;Padi2^−/−^). For the rotarod test, the time spent on rotating rod, latency to fall, was measured in seconds, and for the novel object recognition, test the discrimination index corresponds to percentage ratio between the time exploring the novel object and the total time spent exploring both objects. Means ± SEM are shown, WT n = 8, Padi2 het n = 11, Padi2 fKO n = 11 (^∗^p < 0.05, one-way ANOVA), control (Ct) n = 10, and Padi2 cKO n = 10 (^∗^p < 0.05, Mann-Whitney test). (D) Representative electron microcopy (EM) images for WT and Padi2 fKO corpus callosum. Scale bar, 1 μm. (E and F) Scatterplot of g-ratio as a function of axon diameter (μm) (F) and graph plot for the g-ratio distribution across different axon diameters (E) in WT (n = 4) and Padi2fKO (n = 4). (G) Percentage of myelinated axons present in the corpus callosum of WT and Padi2 fKO. Means ± SEM are shown, n = 4; ^∗^p < 0.05, Mann-Whitney test.

Given the transient decrease in OLs upon *Padi2* cKO in the OL lineage in the spinal cord and the targeting of myelin proteins by PAD2, we investigated whether *Padi2* cKO mice presented a motorical phenotype. Interestingly, *Padi2* cKO mice exhibited impairment in motor coordination and balance as assessed by decreased latency to fall in the rotarod test (Figure 4A). Moreover, we observed similar impairments in the full *Padi2* KO (*Padi2* fKO), in which exon 1 of *Padi2* is deleted in all cells (Raijmakers et al., 2006). In addition, we found an increase in the number of slips on the beam test in the *Padi2* cKO mice, strengthening the motor impairment phenotype (Figure 4B). Interestingly, when testing the *Padi2* cKO mice for cognitive functions, these mice also performed worst in the novel object recognition test, displaying a decrease in the discrimination index that provides an indication of recognition memory (Figure 4C).

To further investigate the mechanisms underlying the impact of PAD2 on motor and cognitive functions, we analyzed the myelin structure of the *Padi2* fKO mice and respective controls using electron microcopy (EM). We found a significant decrease in the g-ratio of small-diameter axons and an increase in the g-ratio of axons with 0.6–0.9 μm diameter (Figures 4D–4F). Moreover, the percentage of myelinated axons in the corpus callosum was decreased in *Padi2* fKO mice compared with controls (Figure 4G). As such, our results indicate that PAD2 plays a role initially for efficient OL differentiation in the spinal cord and later on in myelination in the corpus callosum, which is reflected in the motoric and cognitive defects occurring upon its ablation.

### PAD2 Is Present in Both the Nucleus and Cytoplasm in OL Lineage Cells

**Figure 5:**
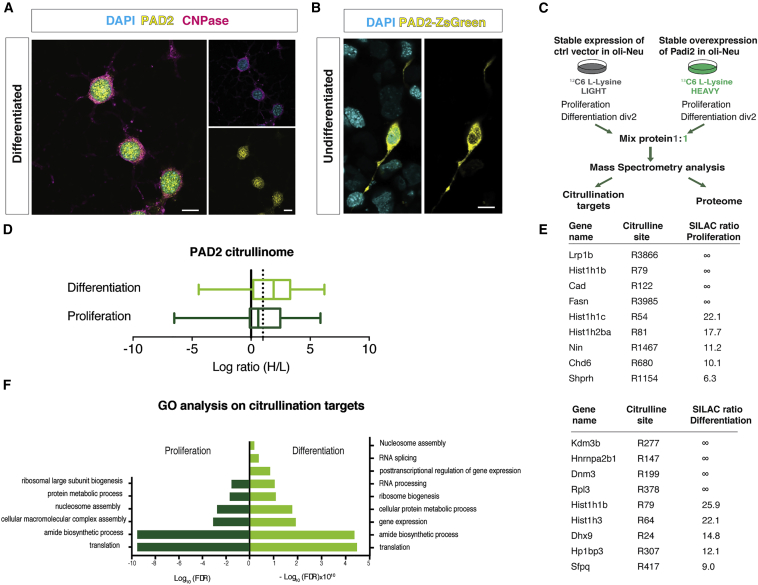
Padi2 Overexpression Unveils Citrullination Targets (A) Immunocytochemistry in 2 day differentiated Oli-neu for PAD2 (yellow) and CNPase (magenta) depicting PAD2 in the nucleus and cytoplasm of cells. Scale bar, 20 μm. (B) Transfection of Oli-neu cells with Padi2 fused with ZsGreen (depicted in yellow) shows the PAD2-ZsGreen both in the cytoplasm and in the nucleus. Scale bar, 20 μm. (C) Schematic representation of the strategy used to determine the citrullination targets by means of SILAC followed by mass spectrometry. Control and overexpressing Padi2 cell lines are fed with ^12^C (light) and ^13^C (heavy) media, respectively. Proteins from proliferating and 2 days differentiating cells are collected from both light and heavy media and mixed 1:1 for further mass spectrometry analysis. (D) Boxplot representing the fold change of the identified citrullination targets in Oli-neu cells in both proliferation and differentiation conditions. Ratios are represented as log_2_(H/L ratio), and PAD2-mediated citrullination targets were considered above a threshold of 1 (dashed line). (E) Table representing top citrullination targets of PAD2 in proliferation and differentiation conditions. ∞ represents proteins for which only peptides with heavy labeling were detected. (F) Gene Ontology (GO) analysis for biological processes on the PAD2 citrullinated proteins. The most significant categories (±log_10_[false discovery rate]) were plotted. See also Figure S2 and Tables S1 and S4.

To disclose the possible mechanisms of action underlying the observed *in vitro* and *in vivo* effects, we further investigated the cellular location of PAD2 in OL lineage cells. Despite that PAD4 is the only one displaying a nuclear localization signal (NLS), PAD2 has recently been detected in the nuclei of mammary epithelial cells (Cherrington et al., 2010) and has been found to inhibit transcription through RNA polymerase (Pol) II citrullination in breast cancer cell line T47D (Sharma et al., 2019). Furthermore, PAD2 was able to citrullinate nuclear histone H3R26 in MCF-7 breast cancer cells (Zhang et al., 2012). Remarkably, immunocytochemistry analysis showed the presence of PAD2 protein both in the nucleus and the cytoplasm of differentiated Oli-neu (Figure 5A) and in primary OPCs (MBP negative) and OLs (MBP positive) (Figure S2E). In order to confirm the localization of PAD2 in the nucleus of OPCs, we transfected Oli-neu cells with the fusion vector *Padi2*-ZsGreen. We observed the presence of the green fusion protein both in the nucleus and cytoplasm of these cells (Figure 5B), indicating that PAD2 can indeed be nuclear in OL lineage cells and might thereby regulate transcription through epigenetic mechanisms.

### PAD2 Citrullinates Nuclear Proteins

To determine what could be the molecular targets of PAD2 in both nuclei and cytoplasm, we generated a stable *Padi2*-overexpressing Oli-neu cell line, in which citrullination activity was greatly enhanced when tested in an antibody-based assay for PAD activity (ABAP) (Zendman et al., 2007) (Figure 7C) and performed quantitative proteomics by applying stable isotope labeling of amino acids in cell culture (SILAC) followed by mass spectrometry (Ong et al., 2002). SILAC relies on the incorporation of a light (^12^C) or a heavy (^13^C) isotopic version of lysine into proteins of control and overexpressing *Padi2* cell lines, respectively. The proteomes of fully light (L) and heavily (H) labeled cells were mixed 1:1, and the relative dissimilarities in the proteome citrullination target sites of these cell populations were determined and quantified using mass spectrometry (Figure 5C; STAR Methods). Interestingly, we observed a striking difference in the number of citrullination targets and ratios between proliferating and differentiating Oli-neu cells (Figure 5D). In the proteome of proliferating Oli-neu, we found up to 151 citrullination sites, of which 81 were upregulated (H/L SILAC ratio higher than 1.5) with *Padi2* overexpression. In contrast, up to 500 citrullination sites were identified in differentiating Oli-neu cells, of which 347 were upregulated upon *Padi2* overexpression (Figure 5D; Table S1). These observations indicate that OLs have an intracellular environment that is more permissive to PAD2 activity than OPCs.

We also uncovered citrullination sites arising only with *Padi2* overexpression (no H/L SILAC ratio with peptides detected only in heavy proteomes; Figure 5E; Table S1). Consistent with our previous observations on the localization of PAD2 in the OL lineage (Figures 4A and 4B), PAD2 targets were not only cytoplasmic, such as ribosomal proteins and myelin proteins (such as HSPA8, CNP and RAB1, ENO1 and EEF1A; Jahn et al., 2009), but also nuclear (Figure 5E; Table S1). Chromatin components and modulators such as histone H1 (HIST1H1B), histone H2b (HIST1H2BA), histone H3 (HIST1H3), the histone demethylase KDM3B, and the chromodomain helicase DNA binding protein 6 (CHD6), among others, were identified to be citrullinated by PAD2 at specific arginines (Figure 5E). We also observed citrullination of several RNA binding proteins, consistent with the abundance or arginines in their RNA binding motifs (Figure 5F). Gene Ontology (GO) analysis indicated that proteins citrullinated by PAD2 are involved in translation, ribosomal biogenesis, and nucleosome assembly in both proliferating and differentiating conditions and RNA splicing, RNA processing, and posttranscriptional regulation of gene expression in differentiating conditions (Figure 5F; Table S4). Thus, PAD2 activity is not circumscribed to myelin and cytoplasmic proteins but is also relevant to nuclear processes.

### PAD2 Protein-Protein Interaction Network Is Enriched in Cytoplasmic and Myelin Proteins

**Figure 6:**
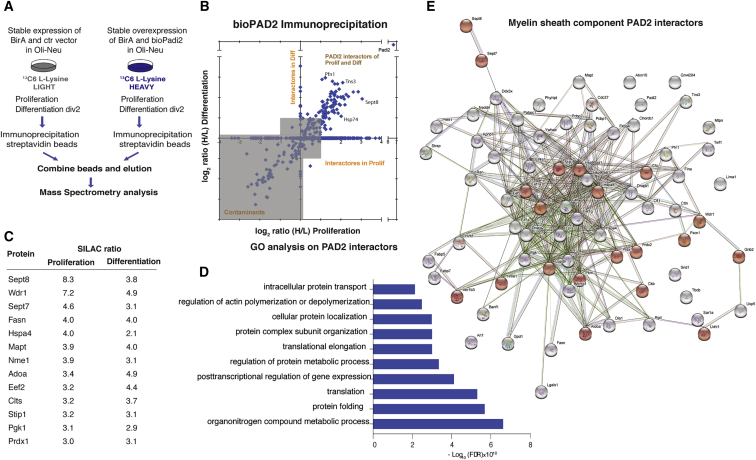
PAD2 Protein Interactors in Oli-neu Cells (A) Schematic representation of the strategy used to uncover PAD2-interacting proteins by means of SILAC followed by mass spectrometry. Control cells (expressing biotin ligase BirA and empty vector) and biotin-tagged *Padi2* (bio*Padi2*) cell lines (expressing BirA and the biotin-tagged *Padi2*) are fed with ^12^C (light) and ^13^C (heavy) media, respectively. Immunoprecipitation (IP) with streptavidin beads is performed on the same amount of proteins collected from control or bio*Padi2* cells. After IP, the streptavidin beads from the two conditions are mixed are analyzed using mass spectrometry. (B) Graph plot representing all detected immunoprecipitated proteins by mass spectrometry in both proliferating and upon 2 days differentiation of Oli-neu cells. Ratios are represented as log_2_(H/L ratio) and PAD2-interacting proteins were considered for analysis when displaying a threshold above 1 and detected in both proliferation and differentiation (all targets in the upright quadrant). (C) Short list of the top ten PAD2-interacting proteins that were previously shown in the myelin proteome. (D) GO analysis for biological processes on the PAD2 interacting proteins. The most significant categories (+/− Log10 (false discovery rate)) were plotted. (E) Network analysis of all PAD2 interactors, proteins found on the cellular component GO analysis for the myelin sheath are highlighted in red. See also Tables S2 and S4.

Our data suggest that PAD2 might be involved in different biological cell processes occurring both in the nucleus and in the cytoplasm. To further explore the PAD2 role in OL lineage cells, we identified the PAD2 protein interactors by pulling down biotin-tagged PAD2. Control (expressing biotin ligase, BirA, and empty vector) and biotin-tagged *Padi2* (bio*Padi2*) Oli-neu cell lines (expressing BirA and the biotin-tagged *Padi2*) were fed with ^12^C or ^13^C isotopes, respectively, immunoprecipitated with streptavidin beads, and combined for further mass spectrometry analysis (Figure 6A). As expected, PAD2 was the protein enriched the most (Figure 6B). We uncovered up to 74 shared PAD2 protein interactors in proliferating and 2 days differentiated Oli-neu cells (Figures 6B–6E; Table S2), including nuclear proteins such as elongator protein complex 1 (ELP1). Nevertheless, the number of interactors was much smaller than citrullination targets, suggesting that most of the PAD2 interactions leading to citrullination are transient and cannot be captured. Yet, GO analysis of the biological processes showed common categories with the GO analysis of the PAD2 citrullination targets, such as translation and posttranscriptional regulation of gene expression (Figure 6D), suggesting once again a role for PAD2 in these processes. Interestingly, one of the categories in the GO analysis, the intracellular protein transport, comprises RAN and KPNB1, two key regulators for active nuclear transport, which suggests that these proteins can be involved in the PAD2 nuclear-cytoplasmic shuttling (Table S2). When performing GO analysis for the cellular component, we also have identified a myelin sheath component of PAD2 interactors (Figure 6E, myelin sheath partners highlighted in red; Table S4). Furthermore, comparison with a previously published myelin proteome (Jahn et al., 2009) uncovers additional myelin proteins that interact with PAD2, such as WDR1 and FASN, among others (Figure 6E; Table S2). Thus, although interactions of PAD2 with nuclear proteins might be more transient, we observed several myelin proteins interacting with this enzyme.

### Histone Citrullination by PAD2 Is Associated with Regulation of OL Differentiation Genes

**Figure 7:**
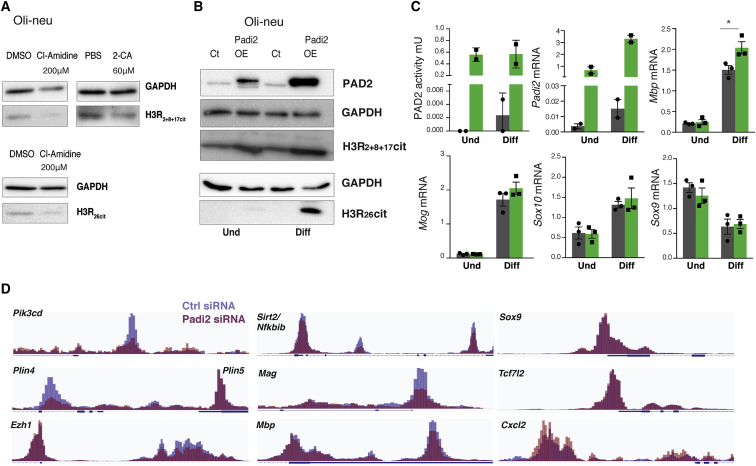
PAD2 Citrullinates Histones and Regulates the Expression and Chromatin Accessibility of Genes Involved in OL Differentiation (A) Western blot for H3R2+8+17cit and H3R26cit upon treatment of proliferating Oli-neu cells with either DMSO or Cl-amidine 200 μM for 2 days. GAPDH signal is an internal loading control. Images represent n = 2. (B) Western blot analysis for PAD2, histone H3R2+8+17cit, and H3R26 on *Padi2*-overexpressing cell line versus control. GAPDH was used as a loading control. Representative images of n = 3. (C) Analysis of Oli-neu cell line overexpressing *Padi2* or scramble control. *Padi2*-overexpressing cells display higher PAD activity, as assessed using the ABAP kit assay, and high mRNA *Padi2*, as assessed using qRT-PCR. Comparative gene expression gene analysis of proliferating and undifferentiated (Und) Oli-neu cells and 2 days differentiated Oli-neu cells (Diff) overexpressing *Padi2*, n = 3 (*Mbp*, *Mog*, *Sox10*, and *Sox9*). Means ± SEM are shown; ^∗^p < 0.05, two-tailed t test. (D) IGV genome browser overlay views depicting chromatin accessibility (assessed using ATAC-seq) near transcription start sites, in Oli-neu cells transfected with control (Ctrl) siRNAs (blue) and siRNA against Padi2 (red) (n = 3, samples pooled for visualization, same scale for Ctrl siRNA and Padi2 siRNA). See also Tables S3 and S4.

Because we found PAD2 in the nucleus of OL lineage cells and identified several chromatin-associated proteins as targets of PAD2, we investigated whether citrullinated nuclear proteins, such as histones, are present in OL lineage cells using an orthogonal technique. Indeed, western blot analysis of histone citrullination showed the presence of histone H3R_26_Cit and histone H3R_2+8+17_Cit in proliferating Oli-neu cells (Figure 7A). Treatment with 200 μM of the pan PAD inhibitor Cl-amidine or 60 μM of 2-CA led to a reduction on these modifications (Figure 7A). We also observed increases of histone H3R_2+8+17_Cit and Histone H3R_26_Cit in the *Padi2*-overexpressing cell lines, indicating that PAD2 is mediating histone H3 citrullination and thus acts as an epigenetic regulator in OL lineage cells (Figure 7B). Although the expression of *Mog*, *Sox10*, and *Sox9* was not altered upon *Padi2* overexpression, we observed upregulation of *Mbp* in differentiating cells (Figure 7C), corroborating their downregulation upon PAD2 inhibition or *Padi2* knockdown (Figure 2). Thus, histone citrullination might be one of the mechanisms by which PAD2 regulates the transition of OPCs to NFOLs.

### *Padi2* Knockdown Results in Reduced Accessibility in OL Differentiation Genes

To further examine the possible role of PAD2 in transcriptional regulation and the chromatin structure, we investigated if the knockdown of *Padi2* would affect the chromatin accessibility. We performed assay for transposase-accessible chromatin using sequencing (ATAC-seq; Buenrostro et al., 2013) to identify changes in accessibility of regulatory regions in differentiating Oli-neu upon knockdown of *Padi2* with siRNAs (Figure 7D). Interestingly, we observed a general reduction in accessibility upon knockdown of *Padi2* (Figures 7D and S3A; Table S3). We further investigated accessibility of OL enhancers and promoters, presenting H3K27ac in Oli-neu cells (see STAR Methods) or H3K27ac and/or H3K4me3 in OPCs (Lu et al., 2016). Indeed, we observed decreased chromatin accessibility in regulatory regions of genes such as *Pik3cd*, *Plin4*, and *Ezh1*. Importantly, we also observed such reduction in accessibility in important regulators of OL myelination, including *Sirt2*, *Mag*, and *Mbp* (Figure 7D) and in *Plp1*, *Sox10*, and *Mog*, although to a minor extent (Figure S3A). Accordingly, GO analysis indicated an enrichment in regulatory regions of genes involved in lipid metabolism (Figure S3B; Table S4). Nevertheless, PAD2 appears to target specific regulatory regions, because we did not find regulation of chromatin accessibility at regulatory regions of other genes modulating OL differentiation, such as *Sox9*, *Tcf7l2* (Figure 7D), *Id1*, and *Olig2* (Figure S3A). Interestingly, we also find a cohort of genes in which accessibility of their regulatory regions was instead increased by Padi2 knockdown, including *Cxcl2* (Figure 7D), *Hspb8*, and *Gabra6* (Figure S3A). GO analysis indicated involvement in the processes of chemotaxis, Polycomb activity, and apoptosis, among others (Figure S3B; Table S4). As such, recruitment of PAD2 to specific regulatory regions in OLs regulates their accessibility, which will also ultimately modulate transcriptional output, as observed for *Mog*, *Mbp*, and *Sox10*.

## Discussion

The formation of myelin is a fundamental biological process that encompasses subsequent steps of OPC specification, differentiation, and myelin sheath formation and wrapping of axons. Our study highlights PAD2 as an important player in these last two steps by (1) facilitating OPC differentiation and influencing the OL epigenetic landscape through histone citrullination and (2) interacting with and mediating the citrullination of several myelin protein components. Although *Padi2* expression in OLs and its occurrence in myelin were described before (Wood et al., 2008), we report its presence in OPCs and the dramatic upregulation of *Padi2* in the transition from OPCs to NFOLs and its function facilitating OL lineage progression. To uncover PAD2 mechanisms of action, we further determined the citrullination targets and the binding partners of PAD2. Our data revealed proteins citrullinated by and interacting with PAD2, both located in the nuclear and cytoplasmic compartments. We also demonstrate, using different *Padi2* KO mouse lines, that PAD2 is important for OL development and motoric and cognitive behavior. Our findings indicate that PAD2-mediated citrullination is required for efficient OL development, not only acting as a myelin modulator, through MBP citrullination, but also displaying other functions in the cytoplasm and nucleus, for instance as an epigenetic modulator. Additional mechanisms underlying PAD2 actions in OPC differentiation remain to be investigated.

Using an unbiased proteomics methodology based on SILAC followed by mass spectrometry, we revealed hundreds of citrullination protein targets and the respective arginine locations, some of those in core histones such as HIST1H1, HIST1H2, and HIST1H3. PAD2-mediated H3R_26_Cit has been shown to lead to local chromatin decondensation and targeted transcriptional activation in breast cancer cells (Guertin et al., 2014). In a similar manner, when we knocked down *Padi2*, we observed a general decrease in chromatin accessibility, as revealed by ATAC-seq. Furthermore, upon PAD inhibition or *Padi2* knockdown, we observed a decrease in the pro-differentiation and myelin genes such as *Mbp*, *Mog*, and *Sox10*, suggesting that PAD2 might be locally enhancing transcription through histone H3 citrullination.

Interestingly, we detected HIST1H1C citrullination on arginine (R) R54 in our cells. This modification was previously described to induce global chromatin decondensation in induced pluripotent stem (iPS) cells (Christophorou et al., 2014) and to be specifically mediated by PAD4, which suggests that there is an overlap of the substrates of PAD2 and PAD4. Moreover, H3R_2+8+17_Cit was also induced upon *hPadi4* overexpression (Christophorou et al., 2014). hPAD2 and hPAD4 were shown to exhibit different substrate preferences and hPAD4 had more pronounced substrate specificity, in both Cos-1 and HEK cells or by adding recombinant hPADs to cell lysates (Assohou-Luty et al., 2014). Additional hPAD2 targets were disclosed by overexpressing hPAD2 in HEK cells using a different technology (Lewallen et al., 2015). Although these are targets for hPAD2 in different human cell types, we observed many common targets with our mouse OL lineage cells, such as ribosomal proteins and splicing factors. Thus, PAD2 might have a widespread and undetermined role in basic cellular processes such as mRNA splicing and translation.

Differentiated Oli-neu cells displayed considerably more citrullination targets, higher citrullination activity in basal conditions with the ABAP kit, and a more pronounced increase in H3R_2+8+17_Cit and H3R_26_Cit upon *Padi2* overexpression, suggesting that PAD2 activity can be enhanced upon differentiation. In agreement, most of PAD2 effects are more noticeable upon OPC differentiation. Of note, PAD activity can also be context dependent, as these enzymes need a high concentration of calcium to become active, and calcium signaling has been shown to be fundamental for OPC migration, differentiation, and myelination (Pitman and Young, 2016). Although several citrullination targets found in proliferating and 2 days differentiated Oli-neu were common and/or showed additional citrullinated arginine in differentiation, they did not overlap completely, indicating that there is a preference for the targeted arginine between these conditions that could be due, for instance, to differences in binding partners. Likewise, not all proteins found to interact with PAD2 were citrullinated, and vice versa. Nevertheless, GO analysis for the biological processes on the PAD2 interactors also revealed categories similar to the citrullination targets, such as translation and posttranscriptional regulation of gene expression, reinforcing once again a putative role for PAD2 in these processes.

Mice overexpressing *Padi2* under the *Mbp* promoter display abnormal myelination with structural changes of the myelin (Musse et al., 2008). This effect was attributed to the increased citrullination of MBP and also of histones that would ultimately cause OL apoptosis (Musse et al., 2008). However, a striking finding in our study is the abundance of myelin component proteins within the interactors of PAD2, which might also mediate the effect of PAD2 on myelin when overexpressed (Musse et al., 2008). Citrullination of myelin proteins is naturally occurring, and thus normal citrullination levels must certainly have a physiological function. 2′,3′-Cyclic-nucleotide 3′-phosphodiesterase (CNPase), which has an important role in the generation of cytoplasmic channels in OLs (Snaidero et al., 2017), displayed PAD2-mediated citrullination of several arginines (Table S1). It is possible that hypocitrullination of CNPase upon knocking out PAD2 could lead to disruption of cytoplasmic channels and ultimately to the observed defects on myelination in our *Padi2* fKO mice (Figure 7). Further investigation would be necessary to determine which citrullination targets of PAD2 in the myelin could be responsible for these defects.

We found that PAD2 absence in OL lineage cells caused decreased OL cell density in the spinal cord in juvenile mouse and a decrease in the number of myelinated axons in the corpus callosum of adult mice that were reflected in motor and cognitive dysfunctions. Because *Pdgfra* and *Padi2* can also be co-expressed in other non-CNS cells, such as Schwann cells, the impairment on motor functions could also be due to the absence of PAD2 in peripheral cells. However, overexpression of *Padi2* did not have any effect on the peripheral myelin (Musse et al., 2008), and the electron microcopy and OL density analysis in the brain and spinal cord (Figure 7) indicated a phenotype in OLs. In addition, the cognitive behavior test also supports a CNS-specific PAD2 effect.

Analysis of single-cell RNA-seq data (Figure S1) suggested that PAD2 is the only member of the PAD family robustly expressed in the OL lineage. Although PAD4 was identified by immunohistochemistry in the myelin (Wood et al., 2008), we could not detect *Padi4* mRNA in any OL lineage cell tested, nor it was identified in the myelin proteome (Jahn et al., 2009). However, we did detect very low mRNA levels of *Padi3* and *Padi1* in Oli-neu cells and found it expressed in very few cells in OPCs or OLs collected from P90 brains (data not shown). As such, we cannot rule out that these PADs have a function in OL lineage progression and that compensatory mechanisms might be operational upon PAD2 knockdown, KO, or inhibition, particularly as PADs can have overlapping substrates.

PAD2 has been long investigated in the context of MS because MBP hypercitrullination is a hallmark of the disease (Yang et al., 2016). Our study demonstrates that PAD2-mediated citrullination is also required for OL lineage progression and myelination and uncovers several citrullination targets in the OL lineage. Thus, targeted activation of PAD2 in the OL lineage might be beneficial in the context of remyelination in diseases as MS.

## STAR★Methods

### Key Resources Table

**Table:** 

REAGENT or RESOURCE	SOURCE	IDENTIFIER
**Antibodies**		

Chicken polyclonal anti-GFP	abcam	ab13970; RRID:AB_300798
Mouse monoclonal anti-CC1 (anti-APC)	Millipore	OP80; RRID: AB_446161
Mouse monoclonal anti-CNP	abcam	ab6319; RRID:AB_2082593
Rat monoclonal anti-MBP (ICC)	abcam	ab7349; RRID:AB_305869
Mouse monoclonal anti-MBP (Western)	Covance	SMI99; #808402 RRID:AB_10120129
Rabbit polyclonal anti-PAD2	ProteinTech	12110-1-AP; RRID:AB_2159475
Rabbit polyclonal anti-cleaved Casp3	Cell Signaling Technology	9661; RRID:AB_2341188
Rabbit polyclonal anti-GFAP	Dako	Z0334; RRID:AB_10013382
Rabbit monoclonal anti-GAPDH	Cell Signaling Technology	5174S; RRID:AB_10622025
Rabbit polyclonal anti- H3Cit2+8+17	abcam	ab5103; RRID:AB_304752
Rabbit polyclonal anti-H3Cit26	abcam	ab19847; RRID:AB_873856
Mouse monoclonal H3K27ac	Millipore	17-683; RRID:AB_1977529
Mouse monoclonal anti-A2B5	Miltenyi Biotec	Clone 105HB29, 130-093-582; RRID:AB_10827602
Mouse Monoclonal anti-β-ACTIN	Sigma	Clone AC15, #A5441; RRID:AB_476744
Mouse monoclonal anti-MBP (Western)	Covance	SMI99; RRID:AB_10120129
Rat monoclonal anti-CD140a	BD Bioscience	Clone APA5, #562777; RRID:AB_2737788
rat anti-mouse IgM beads	Miltenyi	130-047-302; RRID:AB_244359

**Experimental models: Organisms/Strains**		

Mouse: Pdgfrα-CreERT	The Jackson Laboratory	B6N.Cg-Tg(Pdgfra-CreERT)467Dbe/J; RRID:IMSR_JAX:018280
Mouse: Pdgfrα-Cre	The Jackson Laboratory	C57BL/6-Tg(Pdgfra-cre)1Clc/J; RRID:IMSR_JAX:013148
Mouse: Pdgfrα-H2BGFP	Philippe Soriano	B6.129S4-Pdgfra^tm11(EGFP)Sor^/J; RRID:IMSR_JAX:007669
Mouse: RCE: loxP	Gord Fishell, Harvard Medical School	Gt(ROSA)26Sor^tm1.1(CAG-EGFP)Fsh^ in CD1 background; RRID:MMRRC_032037-JAX
Mouse: *Padi2*^*tm1a*(KOMP)Wtsi^ knockout first (promoter driven)	UC Davis KOMP Repository	C57BL/6N-A^tm1Brd^*Padi2*^*tm1a*(KOMP)Wtsi^; RRID:IMSR_KOMP:CSD29542-1a-Wtsi
Mouse: Flp deleter	The Jackson Laboratory	B6; SJL-Tg(ACTFLPe)9205Dym/J; RRID:IMSR_JAX:003800
Mouse: C57BL/6-PAD2-KO	Kerri Mowen, Scripps Research Institute	N/A

**Experimental models: cell line**		

Oli-neu	Jacqueline Trotter, Johannes Gutenberg University, Germany	RRID:CVCL_IZ82
Oli-neu + Padi2_Zc_Green	This paper	N/A
Oli-neu + pB-CAG-Ctr + PBase	This paper	N/A
Oli-neu + pB-CAG-Padi2 + PBase	This paper	N/A
Oli-neu + pB-CAG-BirA + PB-CAG-IRES-GFP + pBase	This paper	N/A
Oli-neu + pB-CAG-BirA + pB-CAG-*bioPadi2*-IRES-GFP + PBase	This paper	N/A

**Recombinant DNA**		

pZsGreen1-N1	Clontech	Cat#632448
pDEST HYGRO	Christophorou et al., 2014	N/A
PBase	Christophorou et al., 2014	N/A
pCAG-DEST-Ctr	Christophorou et al., 2014	N/A
pCAG-Padi2-Hygro	This paper	N/A
pCAG-bioPadi2-IRES-GFP	This paper	N/A
pCAG-IRES-GFP	This paper	N/A
PB-CAG-eGFP	Chen and LoTurco, 2012	N/A
pCALNL-IRES-GFP	Dra. Ursula Wyneken, Universidad de los Andes, Chile	N/A
pEF1aFlagbio (FLBIO)-puro	Stuart Orkin, Harvard Medical School, US (Kim et al., 2009)	N/A
pEF1aBirAV5-neo	Stuart Orkin, Harvard Medical School, US (Kim et al., 2009)	N/A

**Oligonucleotides**		

ON-TARGETplus Non-targeting siRNA #1	Dharmacon GE Healthcare	D-001810-01-20
mouse *Padi2* sense: 5′ CGUACGUGAUGGAGA GGCAUU 3′	This paper	N/A
mouse *Padi2* antisense: 5′ UGCCUCUCCAUCA CGUACGUU 3′	This paper	N/A
Primers for genotyping, see Table S5	This paper	N/A
Primers for qPCR, see Table S5	This paper	N/A

**Critical Commercial Assays**		

Neural tissue dissociation kit (P)	Miltenyi	130-092-628
miRNeasy mini kit	QIAGEN	217004
MaxTract High density	QIAGEN	29046
SILAC Advanced DMEM/F12-FLEX kit	Life Technologies/Thermo Fisher Scientific	MS10033
Antibody Based Assay for PAD activity (ABAP) kit	Modiquest Research	MQ17.101-96
High-Capacity cDNA Reverse Transcription Kit	Applied Biosystems	4368813

**Chemicals, enzymes and recombinant Proteins**		

Gateway® LR Clonase® II Enzyme mix	Invitrogen, ThermosFisher Scientific	11791020
Gateway® BP Clonase® II Enzyme mix	Invitrogen, ThermosFisher Scientific	11789020
Poly-L-lysine	Sigma	P4707
Fibronectin	Sigma	F1141
NeuroBrew	Miltenyi	Miltenyi 130-093-566
bFGF	Peprotech	100-18B
PDGF-AA	R&D	520-BB-050
T3	Sigma	T6397
L-thyroxine	Sigma	89430
N2 supplement	Life Technologies/Thermo Fisher Scientific	17502048
Accutase solution	Sigma	A6964
Erb inhibitor (PD174265)	St Cruz	sc-204170
Cl^−^Amidine	Millipore	506282
Cl^−^Amidine	Cayman Chem	10599
2-chloroacetamidine	Sigma	C0267
Lipofectamine® 2000	Invitrogen, ThermosFisher Scientific	11668019
Hygromycin B Gold	InvivoGen	Ant-hg-1
Fast SYBR® Green Master Mix	Applied Biosystems	4385616
Tamoxifen	Sigma	T5648
ECL™Prime Western Blotting Detection Reagent	GE Heathcare	RPN2236
Supersignal west femto	ThermoFisher Scientific	34095
Streptavidin beads, DB Myone Streptavidin T1	Invitrogen, ThermosFisher Scientific	65601
Protease Inhibitors	Sigma	11873580001
Phenol:Chloroform:Isoamyl (25:24:1)	Invitrogen, ThermosFisher Scientific	15593031
Nextera Tn5 Transposase	Illumina	#FC-121-1030

**Deposited data**		

ChIP seq	This work	GEO: GSE115929

**Software and algorithms**		

Cytoscape	Shannon et al., 2003	http://www.cytoscape.org/; RRID:SCR_003032
Cell profiler	Broad Institute, Cambridge, MA	RRID:SCR_007358
GraphPad Prism 6	www.graphpad.com	www.graphpad.com; RRID:SCR_002798
Mass Spectrometry, Ver. 1.3.0.5	MaxQuant	RRID:SCR_014485
Fiji (ImageJ), Ver. 1.0	https://fiji.sc	RRID:SCR_002285
PeakAnalyzer	Salmon-Divon et al., 2010	RRID:SCR_001194
Bowtie2	Langmead et al., 2009	RRID:SCR_005476
Cutadapt 1.9.1	Martin, 2011	RRID:SCR_011841
SAMtools	Li et al., 2009	RRID:SCR_002105
MACS2	Zhang et al., 2008	https://github.com/taoliu/MACS/, RRID:SCR_013291
Picard 1.126	Broad Institute. “Picard.” (2014).	http://broadinstitute.github.io/picard/; RRID:SCR_006525
bedtools.2.17	Quinlan and Hall, 2010	RRID:SCR_006646
Deeptools2	Ramírez et al., 2016	https://deeptools.readthedocs.io/en/develop/; RRID:SCR_016366

### Contact for Reagent and Resource Sharing

Further information and requests for resources and reagents should be directed to and will be fulfilled by the Lead Contact, Goncalo Castelo-Branco (goncalo.castelo-branco@ki.se).

### Experimental Models and Subject Details

#### Transgenic Mice Strains

All mice were maintained under a pathogen-free environment at the animal facility. Animals were used in adult stage, between 10-12 weeks old and both genders were included. The following light/dark cycle was used: dawn 6.00-7.00; daylight 07.00-18.00; dusk 18-00-19.00; night 19.00-06.00. A maximum of 5 adult mice per IVC-cage of type II Allentown. Breedings were done with 1 male and up to 2 females. All experimental procedures were performed following the guidelines and recommendations of local animal protection legislation and were approved by the local committee for ethical experiments on laboratory animals (Stockholms Norra Djurförsöksetiska nämnd in Sweden). The following transgenic mice strains were used:

*Pdgfra*-H2BGFP knock-in mice(Klinghoffer et al., 2002) where H2B-eGFP fusion gene is expressed under the promoter of *Pdgfra. Pdgfra*-H2BGFP pups of both genders from postnatal day P1 to P4 were used for primary cultures.
*Pdgfra*-Cre BAC transgenic mice (The Jackson Laboratories, CA, USA), where Cre recombinase is under the control of the *Pdgfra* promoter;
RCE:loxP, an EGFP reporter mice (Gord Fishell, NYU Neuroscience Institute);
*Pdgfra*-CreERT BAC transgenic mice (The Jackson Laboratories, CA, USA)
*Padi2*^*tm1a*(KOMP)Wtsi^ (UCDAVIS KOMP Repository Knockout Mouse Project)
*Actb*:FLPe (The Jackson Laboratories, CA, USA).
All individual mice strains were in a C57BL/6NJ genetic background, at the exception of RCE:loxP-GFP that was in a CD1 background.
*Padi2*^fl/fl^ was generated by breeding *Padi2*^*tm1a*(KOMP)Wtsi^ with Flp deleter mice. *Padi2*^fl/fl^ mice were crossed with RCE:loxP, *Pdgfra*-Cre or *Pdgfra*-CreERT.

PdgfraCre; RCE:loxP and PdgfraCre; RCE:loxP; Padi2 mouse lines were used for qPCR analysis at several ages (postnatal P1-P4, juvenile P21 and adult P60) with both genders included. Behavioral analysis of PdgfraCre; RCE:loxP; Padi2 was performed in 4 month adult and both genders were included. Immunohistochemistry analysis was performed in both juvenile P21 (both genders included) and 4-month old adults (only females).

Tamoxifen (Sigma T5648) was dissolved in corn oil and administrated i.p. in P6 *Pdgfra*-CreERT;RCE:loxP;*Padi2*^fl/fl^ pups at a dose of 2mg/40 g. Brain and spinal cord were subsequently collected at P11. Genders were not confidently determined at this age in these pups.

Use of full PAD2 KO mice was strictly compliant with the guidelines set forth by the US Public Health Service in their policy on Human Care and Use of Laboratory Animals, and in the Guide for the Care and Use of Laboratory Animals. Mice were maintained under a pathogen-free environment at the animal facility of Mount Sinai Medical Center. All procedures received prior approval from the Institutional Animal Care and Use Committee (IACUC). The C57BL/6-PAD2-KO mice were obtained from the Scripps Research Institute (Kerri Mowen’s laboratory) which was originally from Lexicon Genetics (Woodlands, TX). Behavioral analysis was performed in 4 month old mice, both genders were included. EM analysis comprised 7 month old males.

#### Cell Lines

Oli-neu + Padi2_Zc_Green, Oli-neu + pB-CAG-Ctr + PBase, Oli-neu + pB-CAG-Padi2 + PBase, Oli-neu + pB-CAG-BirA + PB-CAG-IRES-GFP + pBase and Oli-neu + pB-CAG-BirA + pB-CAG-*bioPadi2*-IRES-GFP + PBase were all generated in this paper from Oli-neu cell lines donated by Jacqueline Trotter, Johannes Gutenberg University, Germany.

### Methods Details

#### Genotyping of Padi2^fl/fl^ Mice

We have assessed the presence of the floxed cassette and the product of the Cre/CreERT mediated DNA deletion (Figure S2A) by performing genotyping in mouse ear or tail DNA. Mouse ear and tail DNA have Pdgfra positive and negative cells and therefore allowed us to classify mice as WT, hetero or homozygous for the floxed cassette but also for the presence or absence of a PCR product band that occurs only when Cre/CreERT is active (post-cre band). We also confirmed the disruption of Padi2 mRNA, at P4 and P21, by qPCR in Figure S2B.

DNA was extracted by incubating biopsies at 98°C for 1h in 75ul of NaOH25mM/EDTA 0.2mM. The reaction was neutralized by adding 75ul of TrisHCl 40mM pH5.5. After centrifugation for 4min at 4000rpm 2 μL were collected and used in PCR reaction using DreamTaq Green PCR Master Mix 2x (Thermo Scientific K1081) following manufacturer’s instructions. Primers:

Padi2-F:AGTTAGAGGCCAACTGTGTGAGACC
Padi2-ttR: CCAGAAAGGACCCACATTCAAGAGC
Padi2-R: AGACTCTGTGCTGACATTCTCAGGG

#### Behavioral Analysis

For the beam balance test the fine motor coordination and balance was assessed by beam walking assay. Performance on the beam was quantified by measuring the time taken by the mouse to traverse the beam and the number of paw slips that occurred in the process. The beam apparatus consisted of a PVC plastic beam (1.2 cm wide × 100 cm long) located 50 cm above the table top on two poles. A black box was placed at the end of the beam as the finish point. Nesting material and feces from home cages was placed in the black box to attract the mouse to the finish point. A nylon or cotton pad was placed below the beam, to cushion any falls. Mice were trained three times a day for two consecutive days until they were able to cross the beam without hesitation. No data were collected during these sessions. During the test session, the time needed to cross the beam and the number of paw slips were recorded. The beam and box were cleaned of mouse droppings and wiped with 70% ethanol before the next animal was placed on the apparatus.

The novel object recognition task was conducted as described previously (Fortress et al., 2013), and was used to assess the non-spatial hippocampal memory. A mouse was presented with two similar objects during the first session, and then one of the two objects was replaced by a new object during a second session. The amount of time taken to explore the new object provided the index of recognition memory. The task consisted of habituation, training, and testing phases conducted on separate days. During habituation, mice were allowed to freely explore an empty white box (60 cm wide × 60 cm long × 47 cm high) for 5 min/day for two days. Twenty-four hours later, mice were re-habituated (familiarization session) in the same box for 10 min, with two identical objects placed inside, 5 cm away from the walls. Mice were allowed to freely investigate until they accumulated a total of 20 s exploring the objects. Exploration and recognition of the object were determined when the nose of the animal was in contact with an object or directing nose to the object within a defined distance (< 2 cm). Mice were then immediately returned to their home cage. After the familiarization session, the objects and the open field was cleaned with 70% ethanol to minimize olfactory cues. After 1 hour of familiarization session, object recognition was tested, using the same procedure as in training except that one of the familiar objects was substituted with a novel object (cleaned and free of olfactory cues). The position of the novel object (left or right) has to be randomized between each mouse and each group tested. Time spent with each object was recorded using a stopwatch by a trained observer. Mice inherently prefer to explore novel objects; thus, a preference for the novel object indicates intact memory for the familiar object. A discrimination index was calculated according to the following formula: time of novel object exploration/time of novel plus familiar object exploration × 100.

Rotarod behavioral analysis was performed for evaluation of coordination and motor learning. Mice were trained on a Rotarod (Ugo Basile, Comerio VA, Italy) at a constant speed (4 rpm), 120 s per day for 3 consecutive days. Subsequently, test day consisted of three trials on an accelerating rotarod for up to 300 s (starting at 4 rpm accelerating to a final speed of 40 rpm), with mice returned to their home cages for at least 30 min between trials. The time at which each animal fell from the rod was recorded as a measure of motor coordination and repeated-measurements of speed at the time of animal falling were averaged.

#### Electron Microscopy

For EM analysis, deeply anesthetized C57BL/6-PAD2-KO mice (n = 4 per condition) were perfused transcardially using a varistaltic pump (Manostat) with 0.1M PBS (pH 7.4) followed by cold Karnovsky’s fixative (2.0% PFA/2.5% Glutaraldehyde in 0.1M Sodium Phosphate buffer).

Using a McIlwain tissue chopper, coronal brain sections between −2.2 and −2.5 bregma were obtained and corpus callosum tissue was micro-dissected and post-fixed overnight in 2.0% PFA/ 2.5% Glutaraldehyde in 0.1M Sodium Phosphate buffer. The samples were then processed using standard protocols employed by the Stony Brook University Microscopy Core (see below, and Menezes et al. [2014] and Relucio et al. [2009]). The following day (after overnight post-fixation), an additional fixation step was carried out using 2% osmium tetroxide in 0.1M PBS (pH 7.4), after which samples were dehydrated in a graded series of ethyl alcohol. Finally, samples were embedded using Durcupan resin in between two pieces of ACLAR sheets (Electron Microscopy Sciences). Ultrathin sections of 80nm were obtained using a Lecia EM UC7 ultramicrotome and placed on formvar coated slot copper grids (Electron Microscopy Sciences). Uranyl acetate and lead citrate counterstained samples were viewed under a FEI Tecnai12 BioTwinG2 electron microscope. Digital images (minimum 10 per animal, taken at 11,000x) were acquired using an AMT XR-60 CCD Digital Camera system and compiled using Abobe Photoshop software.

G-ratio was determined by dividing the mean axon diameter without myelin by the same axon diameter with myelin. A minimum of 100 healthy axons were considered per animal. To determine the size distribution of myelinated fibers in corpus callosum, diameters of all measured axons were separated into four ranges (0.3-0.6, 0.6-0.9, 0.9-1.2, and >1.2 μm). No axons with diameters smaller than 0.3 μm were considered. Data are expressed as comparison of means between the control and knock-out for each range. Percentage of myelinated axons was determined by placing a centered grid over the image using ImageJ software, and counting whether the axon at each intersection was myelinated or not. Each data point in the graph corresponds to the percentage of myelinated/unmyelinated axons, quantified from 4 animals per each condition (4 wild-type and 4 PAD2 mutant mice). Quantification has been performed by counting at least 100 axons per image, for a total of 10 images per animal. All measurements were acquired on electron microscopy sections images using an ImageJ software. Unpaired t test were performed to assess statistical differences between control and knock out mice for both differences in g-ratio per axon diameter range and percent myelinated axons.

#### Tissue Dissociation

Cells were isolated from postnatal, juvenile, and adult brains from PdgfraCre;RCE:loxP mice, and from juvenile brains from PdgfraCre;RCE:loxP;*Padi2*^+/+^ and PdgfraCre;RCE:loxP;*Padi2^-/-^* mice with Neural (for postnatal) and Adult (for juvenile and adult) Tissue Dissociation kits (P) (Miltenyi Biotec). Cell suspensions were then filtered with 30 μm filter (Partec) and FACS sorted for selection of GFP^+^. FACS sorted cells were collected in Eppendorf tubes, centrifuged at 400xg, resuspended in Qiazol and frozen for further RNA extraction/cDNA synthesis.

#### FACS

GFP^+^ cells were FACS sorted using a BD FACSAria III Cell Sorter B5/R3/V3 system. Cells were collected either in PBS for further RNA extraction processing or in OPC media directly into coated plates. For OPC removal on OL lineage cells from juvenile and adult brains, CD140a (anti-mouse CD140 APC conjugated, BD Bioscience) labeling was performed in tissue-isolated cells and GFP^+^CD140a^+^ cells were excluded. To define the gates for specific CD140a^+^ cells we have performed an additional labeling with APC conjugated IgG (BD Bioscience) as a negative control.

#### Cell Culture

##### Primary OPC Culture

GFP^+^ OPCs were obtained with FACS from P1-P4 brains (mixed genders) from the *Pdgfra*-H2BGFP mouse line. The mouse brains were removed and dissociated in single cell suspensions using the Neural Tissue Dissociation Kit (P) (Miltenyi Biotec, 130-092-628) according to the manufacturer’s protocol. Cells were seeded in poly-L-lysine (O/N) (Sigma P4707) plus fibronectin (1h) (Sigma F1141) coated dishes and grown in proliferation media comprising DMEM/Gmax (life tec 10565018), N2 media (life tec 17502048), Pen/Strep (life tec 15140122), NeuroBrew (Miltenyi 130-093-566), bFGF 20ng/ml (Peprotech 100-18B) and PDGF-AA 10ng/ml (R&D 520-BB-050). For OPC differentiation, cells were left for 2 days in medium without bFGF and PDGF-AA.

##### Oli-neu Cell Culture

Oli-neu cells were grown in poly-L-lysine coated dishes and expanded in proliferation media consisting of DMEM ((life tec 41965062), N2 supplement, Pen/Strep Glu (life tec 10378016), T3 (sigma T6397) 340ng/ml, L-thyroxine (sigma 89430) 400 ng/ml, bFGF 10ng/ml and Pdgf-BB 1ng/ml. For Oli-neu differentiation, media without growth factors was added for two days *in vitro* and in the presence of 1 μM of erb inhibitor (PD174265, St Cruz, ref sc-204170).

##### Rat OPC Cultures

Primary rat OPCs were isolated as reported previously (Liu et al., 2015). Briefly, mixed glia cultures were first derived from rat brain cortex at postnatal day 1. OPCs were selected by the A2B5 antibody followed by purification with the magnetic rat anti-mouse IgM beads (Miltenyi Biotec). Purified OPCs were cultured in the SATO medium supplemented with PDGFA and bFGF, and were induced to differentiate with T3 replacing the growth factors.

Padi inhibition: Oli-neu cells were treated either with Cl^−^Amidine (Millipore 506282 and Cayman Chem 10599) or 2-chloroacetamidine (2CA) (Sigma C0267) at a concentration of 200 μM and 60 μM for one and two days, respectively, in proliferation and differentiation media.

##### SILAC

To generate LIGHT and HEAVY labeled cell lines, Oli-neu cells were grown and expanded in LIGHT (Lysine ^12^C) or HEAVY (Lysine ^13^C) media for at least five passages. The media was prepared with SILAC Advanced DMEM/F12-FLEX kit (Life Tec, MS10033) and contained 0.1% of dialyzed FBS, 10ng/ml bFGF, 10ng/ml PDGF-BB, 400ng/ml of L-thyroxine and 340ng/ml of T3. For the differentiation, growth factors and the FBS were removed.

LIGHT labeled cells lines correspond to all controls, scramble overexpression control and BirA control, while HEAVY labeled cell lines comprise the overexpression of *Padi2* or the biotin-tagged *Padi2*. A control experiment in which control cells were also fed with heavy media and then mixed 1:1 with light control cells was performed in order to confirm that all proteins in heavy conditions were heavily labeled (Figure S2B).

#### Padi2 Overexpression Cell Lines and Knockdown

*Padi2* and Ctr overexpression cell lines were generated by insertion of the following vectors with piggyBac transposon system: pB-CAG-Ctr, pB-CAG-PADI2 for the control and overexpression of Padi2, respectively; pB-CAG-BirA together with pB-CAG-IRES-GFP as a control and pB-CAG-BirA together with pB-CAG-*bioPadi2*-IRES-GFP for the biotin-tagged *Padi2*. BirA and bioPadi2 cDNA were amplified from the plasmids pEF1aBirAV5-neo and pEF1aFlagbio(FLBIO)-puro (Kim et al., 2009) before inserting in piggyback plasmids. The gateway system was used to clone all these vectors in the final piggyback plasmids (Gateway® LR Clonase® II Enzyme mix, Invitrogen/ThermosFisher Scientific, 11791020 and Gateway® BP Clonase® II Enzyme mix, Invitrogen/ThermosFisher Scientific, 11789020). pCAG-IRES-GFP was generated by modifying the donor plasmid (pB-CAG-eGFP) by replacing the eGFP by a IRES-GFP sequence subcloned from the plasmid pCALNL-IRES-GFP). pB-CAG-Ctr, pB-CAG-*Padi2*, pB-CAG-BirA + pB-CAG-IRES-GFP and pB-CAG-BirA + pB-CAG-*bioPadi2*-IRES-GFP were transfected together with piggyBac transposase (pBase) expression vector by lipofection according to the manufacturer’s instructions (Lipofectamine® 2000, Invitrogen 11668019). To select for biotin-tagged *Padi2* and pB-CAG-IRES-GFP vectors cells were FAC sorted for GFP, all the other cell lines express the hygromycin resistance gene in the DNA integrated vectors and were selected and expanded in media containing 200 μgml^−1^ of hygromycin (Hygromycin B gold ant-hg-1, InvivoGen).

For the expression of the fusion *Padi2* plasmid in Oli-neu OPCs, *Padi2* was cloned into the pZsGreen1-N1 plasmid (Clontech, 632448). Oli-neu and primary mouse OPCs were then transfected with these plasmids with Lipofectamine2000 according to the manufacturer’s instructions.

For silencing *Padi2* we used the siDESIGN center from Dharmacon. The following siRNAs were selected and purchased from Dharmacon GE Healthcare: mouse *Padi2*; sense: 5′ CGUACGUGAUGGAGAGGCAUU 3′; antisense: 5′ UGCCUCUCCAUCACGUACGUU 3′ and ON-TARGETplus Non-targeting siRNA #1 (D-001810-01-20).

Knockdown experiments for *Padi2* were performed both in Oli-neu and mouse primary OPCs with Lipofectamine2000, following manufacturer’s instructions. For mouse primary OPCs, upon 4h of adding the lipids:DNA complexes to the cells, media was changed to either proliferation or differentiation and cells were collected two days after. For Oli-neu experiments, upon 4h of adding the lipids:DNA complexes to the cells, media was changed to proliferation for one day and cells were either collected at this point (proliferation condition) or changed to differentiation media one more day (differentiation condition)

#### ABAP

Cells were collected in lysis buffer [20mM Tris-HCl, pH 7.4; 100mM NaCl; 10mM β-mercaptoethanol; 10% glycerol; protease inhibitor complete (Roche)] and sonicated for 5 minutes at high power with 30 s on/off cycles at 4°C. Then the samples were centrifuged at 13000 x g at 4°C for 30 minutes and the supernatant was immediately used for further application. PAD activity was determined with the Antibody Based Assay for PAD activity (ABAP) kit (Modiquest Research, MQ17.101-96) and performed according to manufacturer’s protocol. HRP-conjugated secondary antibody was visualized with a TMB substrate solution [1 mg/ml TMB (Sigma Aldrich); 0.1M sodium acetate, pH 5.2; 0.01% hydrogen peroxide] and the reaction was stopped with sulfuric acid [2M H_2_SO_4_]. The absorbance at 450 nm was determined with a FLUOstar Omega plate reader (BMG Labtech). Human PAD4 enzyme was used as control enzyme activity and was diluted in deimination buffer [40 mM TrisHCl, pH 7.5; 5 mM CaCl_2_; 1mM DTT] with concentrations between 0.002 mU (minimum deimination) and 2.0 mU (maximum deimination) to create a standard curve to correlate activity to the optical density measured at 450 nm.

#### RNA Extraction, cDNA Synthesis, and Quantitative Real-Time PCR (qRT-PCR)

RNA was extracted with the miRNeasy mini kit (QIAGEN, 217004) for the Oli-neu cells or with the miRNeasy micro kit (QIAGEN, 217084) for the primary OPCs and direct tissue isolated cells according to manufacturer’s protocols. Contaminating DNA was degraded by treatment of the samples with RNase-free DNase (QIAGEN, 79254) in column. 0.35-1μg of RNA from each sample was reversed transcribed for 1h with the High-Capacity cDNA Reverse Transcription Kit (Applied Biosystems, 4368813) including RNase inhibitor (Applied Biosystems, N8080119). An RT- control was included for each sample. Both the cDNA and the RT- were diluted 1:5 in RNase/DNase free water.

qPCR reactions were run on a StepOnePlus System (Applied Biosystems) in duplicate and with reverse transcriptase negative reactions to control for genomic DNA. Fast SYBR® Green Master Mix (Applied Biosystems, 4385616) was used according to the manufacturer’s instructions, each PCR reaction had a final volume of 10 μL and 1–2.5 μL of diluted cDNA and RT-. The running conditions were 20 s at 95°C, followed by 40 cycles of 3 s of 95°C and 30 s of 60°C, then 15 s at 95°C, 1 minute at 60°C and 15 s at 95°C. A melting curve was obtained for each PCR product after each run, to control for primer dimers and gene-specific peaks. Random PCR products were also run in a 2%–3% agarose gel to verify the size of the amplicon. Tbp, r18S and Gapdh were run as housekeeping genes. Relative standard curves were generated for each gene to determine relative expression (CT values are converted to arbitrary quantities of initial template per sample). Expression levels were then obtained by dividing the quantity by the value of the geometric mean of the housekeeping genes.

PCR primer sequences used are listed in Table S5.

#### Immunofluorescence

For immunocytochemistry cells were fixed in 4% formaldehyde for 10 minutes, washed in PBS and incubated overnight at 4°C with the primary antibodies anti-CNP (abcam ab6319, 1:200) anti-MBP (Abcam, ab7349, 1:200), anti-PAD2 (rabbit polyclonal; 12110-1-AP; ProteinTech; 1:200), anti-cleaved Casp3 (rabbit, cell signaling #9661S; 1:200) in PBS/0.5%Trition/10% normal donkey serum (Sigma, D9663). Cells were washed with PBS and then incubated for 2 hours with Alexa Fluor-conjugated antibodies (Invitrogen, Alexa Fluor anti-mouse 488 1:1000 and Alexa Fluor anti-mouse 555 1:1000).

For immunohistochemistry mice were perfused at P11 (for tamoxifen-induced *Pdgfra*CreERT;RCE:loxP/*Padi2*^+/+^ and *Pdgfra*CreERT;RCE:loxP;Padi2^−/−^) or at P21 and at 4 months (for *Pdgfra*-Cre;RCE:loxP;Padi2^+/+^ and *Pdgfra*Cre;RCE:loxP;Padi2^−/−^) with PBS followed by 4% PFA. Brains and spinal cords were dissected and post-fixed with 4% PFA for 1h, at 4°C. The tissues were then cryo-protected with a 30% sucrose solution for 24 hours. The tissues were embedded into OCT (Tissue-Tek) and sectioned coronally (20 um thickness). Sections were incubated overnight at 4°C in the following primary antibodies: GFP (Abcam, ab13970, chicken 1:2000) and CC1 (anti-APC; Millipore, OP80, Mouse 1:100), GFAP (rabbit Dako, Z0334; 1:500) diluted in PBS/0.5% Triton/10% normal donkey serum. After washing the section with PBS, secondary Alexa Fluor-conjugated antibodies diluted in PBS/0.5% Triton/10% normal donkey serum were incubated for 2h at room temperature (Invitrogen, Alexa Fluor anti-chicken 488 1:1000 and Alexa Fluor anti-mouse/goat 555 1:1000). Slides were mounted with mounting medium containing DAPI (Vector, H-1200) and kept at 4°C until further microscopic analysis.

#### Microscopy and Quantitative Analysis

To estimate the number of OL (CC1^+^ cells), newly formed/Cre recombined OL (GFP^+^CC1^+^) and Cre recombined astrocytes (GFP^+^GFAP^+^), 2-3 sections per animal (at same positions of the anterior-posterior axis) were analyzed. For each section, images were taken for the entire corpus callosum and dorsal funiculus of the spinal cord using a Zeiss LSM700 Confocal. The total number of CC1^+^ cells and GFP^+^CC1^+^ or GFP^+^GFAP^+^ double positive cells was counted using ImageJ software. The percentage of OL and astrocytes out of total recombined cells at P11 corpus callosum and spinal cord was calculated by estimating the percentage ratio of GFP^+^CC1^+^ out of total GFP^+^cells for OLs and GFP^+^GFAP^+^ out of total GFP^+^cells for astrocytes. For the estimation of OL density in juvenile mice confocal images were averaged in ImageJ using z-projection of the max intensity and then loaded into the open-source software program CellProfiler (Broad Institute, Cambridge, MA), followed by segmentation of images and identification and relating of primary (nuclei) and secondary (CC1 stained) objects using otsu and global manual thresholding respectively. The counting of CC1 positive or negative cells was calculated in region of interest (ROI) using custom mask included in the pipeline, and normalized to the area.

#### Western Blot

Cells were collected in 2x Laemmli buffer [120mM Tris-HCl, pH 6.8; 4% SDS; 20% glycerol] and sonicated for 5 minutes at high power with 30 s on/off cycles at 4°C to shear genomic DNA. Protein concentrations were determined on nanodrop and concentrations were equalized with 2x Laemmli buffer. Bromophenol blue (0.1%) and β-mercaptoethanol (10%) were added to the samples and the samples were boiled at 95°C for 5 minutes to denature the protein. Equal volumes were loaded in a SDS-PAGE for protein separation and transferred (100V for 90’) to a PVDF membrane (GE-healthcare) activated in methanol. The membranes were blocked in blocking solution [TBS; 0.1% Tween 20; 5% milk] for 1 hour at room temperature and incubated overnight with primary antibody (diluted in blocking solution) at 4°C, washed 3 times 10 minutes in TBS-T [TBS; 0.1% Tween 20] and incubated with a horseradish peroxidase (HRP)-conjugated secondary antibody (diluted in blocking solution) for 2 hours at room temperature. Proteins were exposed with ECL Prime western blotting detection reagent kit (GE healthcare) or Supersignal west femto (Thermo Scientific) at a ChemiDoc XRS imaging system (Bio-Rad). Primary antibodies were used against PADI2 (rabbit polyclonal; 12110-1-AP; ProteinTech; 1:300; 1:1000 for tissue), GAPDH (rabbit monoclonal; 5174S; Cell Signaling; 1:1000), H3Cit2+8+17 (rabbit polyclonal; ab5103; Abcam; 1:1000), H3Cit26 (rabbit polyclonal; ab19847; Abcam; 1:1000), MBP (Covance, SMI99, 1:1000) and ACTIN (Sigma, AC15, 1:10, 000). Secondary antibodies were used at a dilution of 1:5000, anti-rabbit (A6667; Sigma), anti-mouse (A4416; Sigma).

#### PAD2 Immunoprecipitation

Proliferating and 2-days differentiated BirA controls and biotin-tagged *Padi2* cells were fixed for 8 min at RT in 1% formaldehyde solution by adding 1/10 volume to cell media of freshly prepared 11% formaldehyde solution (formaldehyde 11%, 0.1M NaCl, 1mM EDTA pH8, 0.5mM EGTA pH8 and 50mM HEPES pH8). Glycine was added (final 0.125M) to quench formaldehyde, rinsed twice in cold PBS and scrapped for subsequent centrifugation at 4000 g for 10min at 4°C. At this point cell pellets can be stored at −80°C. Cell pellets were then resuspended in 1mL of lysis buffer (10mM HEPES-KOH pH7.5, 100mM NaCl, 1mM EDTA, 0.5mM EGTA, 0.1% Na-Deoxycolate, 0.5% Na-lauroylsarcosinate, protease inhibitors) and sonicated. Cell suspension was centrifuged at 14000 g for 10min at 4°C to remove the debris. For the immunoprecipitation, protein concentrations were determined in every sample and equal amounts of protein were used for further streptavidin pull-down. 50 μL of streptavidin beads (DB Myone Streptavidin T1, Invitrogen) per 5mg of protein were added to the protein suspensions and incubated overnight at 4°C. Magnetic stand was used to precipitate the beads. At this step beads from BirA controls and biotin-tagged Padi2 were combined for the following washes: 4 times in wash buffer 1 (50mM HEPES pH7.6, 1mM EDTA, 0.1% Na-Deoxycolate), 2 times in wash buffer 2 (50mM HEPES pH7.6) and 2 times in wash buffer 3 (20 mM ammonium bicarbonate NH_4_HCO_3_). The beads can be stored at −80°C in this step.

#### Mass Spectrometry for Citrullination Targets

##### SDS-PAGE Gel

Extracted proteins were resuspended in Laemmli Sample Buffer, and resolved on a 4%–20% SDS-PAGE. The gel was stained with Coomassie blue, cut into 20 slices and processed for mass spectrometric analysis using standard in gel procedure (Shevchenko et al., 2006). Briefly, cysteines were reduced with dithiothreitol (DTT), alkylated using chloroacetamide (CAA), and finally the proteins were digested overnight with endoproteinase Lys-C and loaded onto C18 StageTips prior to mass spectrometric analysis.

##### Mass Spectrometric Analysis

All MS experiments were performed on a nanoscale EASY-nLC 1000 UHPLC system (Thermo Fisher Scientific) connected to an Orbitrap Q-Exactive Plus equipped with a nanoelectrospray source (Thermo Fisher Scientific). Each peptide fraction was eluted off the StageTip, auto-sampled and separated on a 15 cm analytical column (75 μm inner diameter) in-house packed with 1.9-μm C18 beads (Reprosil Pur-AQ, Dr. Maisch) using a 75 min gradient ranging from 5% to 40% acetonitrile in 0.5% formic acid at a flow rate of 250 nl/min. The effluent from the HPLC was directly electrosprayed into the mass spectrometer. The Q Exactive plus mass spectrometer was operated in data-dependent acquisition mode and all samples were analyzed using previously described ‘sensitive’ acquisition method (Kelstrup et al., 2012). Back-bone fragmentation of eluting peptide species were obtained using higher-energy collisional dissociation (HCD) which ensured high-mass accuracy on both precursor and fragment ions (Olsen et al., 2005).

##### Identification of peptides and proteins by MaxQuant

The data analysis was performed with the MaxQuant software suite (version 1.3.0.5) as described (Cox and Mann, 2008) supported by Andromeda (www.maxquant.org) as the database search engine for peptide identifications (Weidner et al., 1990). We followed the step-by-step protocol of the MaxQuant software suite (Cox et al., 2009) to generate MS/MS peak lists that were filtered to contain at most six peaks per 100 Da interval and searched by Andromeda against a concatenated target/decoy (Elias and Gygi, 2007) (forward and reversed) version of the IPI human database. Protein sequences of common contaminants such as human keratins and proteases used were added to the database. The initial mass tolerance in MS mode was set to 7 ppm and MS/MS mass tolerance was set to 20 ppm. Cysteine carbamidomethylation was searched as a fixed modification, whereas protein N-acetylation, oxidized methionine, deamidation of Asparagine and Glutamine, and citrullination of Arginines were searched as variable modifications. A maximum of two mis-cleavages was allowed while we required strict LysC specificity. To minimize false identifications, all top-scoring peptide assignments made by Mascot were filtered based on previous knowledge of individual peptide mass error. Peptide assignments were statistically evaluated in a Bayesian model on the basis of sequence length and Andromeda score. We only accepted peptides and proteins with a false discovery rate of less than 1%, estimated on the basis of the number of accepted reverse hits.

#### Mass Spectrometry for PAD2 Interactome

##### LC-MS/MS Sample Preparation

The beads were thawed and resuspended in 100μl 25 mM ammonium bicarbonate. The samples were reduced for 1 hour at room temperature by addition of 1 mM DTT, followed by alkylation for 10 minutes in the dark with 5 mM iodoacetamide. The reaction was quenched by the addition of 5 mM DTT. 0.2 μg of Trypsin (sequencing grade modified, Pierce) was added to the samples and digestion was carried out over night at 37°C. After digestion, samples were spun briefly and the supernatant carefully transferred to a glass vial and dried in a SpeedVac and resuspended in 3% acetonitrile, 0.1% formic acid. On third of the original sample was injected for LC-MS/MS analysis.

##### LC-ESI-MS/MS Q-Exactive

Online LC-MS was performed using a Dionex UltiMate 3000 RSLCnano System coupled to a Q-Exactive mass spectrometer (Thermo Scientific). Samples were trapped on a C18 guard desalting column (Acclaim PepMap 100, 75um x 2 cm, nanoViper, C18, 5 μm, 100 Å), and separated on a 50 cm long C18 column (Easy spray PepMap RSLC, C18, 2 μm, 100Å, 75 μmx15cm). The nano-capillary solvent A was 95% water, 5%DMSO, 0.1% formic acid; and solvent B was 5% water,5% DMSO, 95% acetonitrile, 0.1% formic acid. At a constant flow of 0.25 μL min^−1^, the curved gradient went from 2%B up to 40%B in 95 min, followed by a steep increase to 100%B in 5 min.

FTMS master scans with 70,000 resolution (and mass range 300-1700 m/z) were followed by data-dependent MS/MS (35 000 resolution) on the top 5 ions using higher energy collision dissociation (HCD) at 30%–40% normalized collision energy. Precursors were isolated with a 2 m/z window. Automatic gain control (AGC) targets were 1e6 for MS1 and 1e5 for MS2. Maximum injection times were 100ms for MS1 and 150-200ms for MS2. The entire duty cycle lasted ∼2.5 s. Dynamic exclusion was used with 60 s duration. Precursors with unassigned charge state or charge state 1 were excluded. An underfill ratio of 1% was used.

##### Peptide and Protein Identification

The MS raw files were searched using Sequest-Target Decoy PSM Validator under the software platform Proteome Discoverer 1.4 (Thermo Scientific) against a mouse database (Uniprot, downloaded on 140320) and filtered to a 1% FDR cut off.

We used a precursor ion mass tolerance of 10 ppm, and a product ion mass tolerance of 0.02 Da for HCD-FTMS. The algorithm considered tryptic peptides with maximum 2 missed cleavage; carbamidomethylation (C) as a fixed modification; oxidation (M), and heavy SILAC labeled lysines (Label 13C(6) / +6.020 Da (K)) as dynamic modifications.

#### ChIP-Seq

Oli-neu cells were rinsed with cold PBS and cross-linked with 1% formaldehyde (Sigma) for 10 min at room temperature (RT) on a rotating platform. Fixation was quenched with 0.125M glycine for 5 min at RT. Cells were rinsed with cold PBS containing 1x Protease Inhibitors (PI, Sigma, 11873580001) and collected spinning at 1500rpm for 5 min at 4°C. Pellets were resuspended in swelling buffer (PIPES 5mM, KCl 85 mM, Igepal-CA630 1%, PI 1x), incubated 15min on ice followed by a centrifugation of 1300 g for 5min. This step was repeated twice and nuclear pellets were resuspended in lysis buffer (1% SDS, 10 mM EDTA, 50 mM Tris-HCl (pH 8.0) and PI 1x) and incubated for 10 min on ice followed by chromatin sonication using a Bioruptor™ UCD200 (Diagenode), high frequency, 30 s ON/30 s OFF, for 10 min. Protein A/G coated beads (Invitrogen) were washed and resuspended in ChIP dilution buffer (0.01% SDS, 1% Triton, 1.2mM EDTA, 16.7mM TrisHCl pH8, 167mM NaCl and PI 1x). 20μg of chromatin per ChIP was diluted in ChIP dilution buffer and precleared by incubating with 20μl of pre-washed protein A/G coated beads for 1.5h at 4°C on a rotator. Beads were removed and 2μg of the antibody H3K27ac (Millipore, 17-683) was added to the chromatin and incubated overnight at 4°C on a rotator. Incubate 20μl of beads in 1mg/ml of BSA in PBS overnight at 4°C on a rotator. Wash beads in ChIP buffer and add to the chromatin/ab for 1h at 4°C on a rotator. Chromatin/ab/beads were washed 3 times low salt wash buffer (0.1% SDS, 2mM EDTA, 20mM TrisHCl pH8, 1% Triton X-100, 150mM NaCl), once with high salt wash buffer (0.1% SDS, 2mM EDTA, 20mM TrisHCl pH8, 1% Triton X-100, 500mM NaCl), once with LiCl wash buffer (0.25M LiCl, 1% IGEPAL-CA630, 1% deoxycholate, 1mM EDTA, 10mM TrisHCl pH8) and once with TE buffer (10 mM Tris-HCl (pH 7.5), 1mM EDTA). Chromatin was eluted by incubating the beads with elution buffer (1% SDS, 0.1M NaHCO3) for 15 min at RT. Eluted chromatin was reverse cross-linked by adding 10ng/ml of Proteinase K (Thermo Scientific) and 0.1M NaCl. After 2h incubation at 42°C, samples were incubated at 65°C O/N. Following morning, 10ng/ml of glycogen (Invitrogen) and 1 volume of Phenol:Chloroform:Isoamyl (25:24:1, Invitrogen, 15593031) were added and solutions were mixed thoroughly before isolating the DNA with Maxitract High Density Tubes (QIAGEN). 2.5 Volumes of 100% ethanol and 1/10 volume of 3M NaOAc (Sigma) were added to the sample and incubated for 15 min at −80°C. DNA was washed with 70% ethanol and resuspended in nuclease free water.

#### ATAC-Seq

ATAC-seq was performed as previously described (Buenrostro et al., 2013) with minor adaptations. Oli-neu cells (differentiated, siRNA treated as described above) were collected with Accutase solution (Sigma, A6964). 50 000 cells per condition were lysed in lysis buffer (10 mM Tris-HCl, pH 7.4, 10 mM NaCl, 3 mM MgCl2 and 0.1% IGEPAL CA-630) and centrifuged at 500xg at 4°C for 20 minutes. The cells were then resuspended in tagmentation mix (2.5 μL TD buffer, 2.5 μL Tn5 enzyme, Illumina) and the DNA was transposed for 30 minutes at 37°C, where after the DNA was purified using the QIAGEN MinElute kit. After PCR amplification with 7-8 cycles (cycles determined with qPCR) the libraries were sequenced on a HiSeq2500 Illumina sequencer. Three replicates per condition were performed in different days and different passages. Each replicate had >10 million reads with an average of 45 million reads per sample.

#### Bioinformatic Analysis of ChIP-Seq and ATAC-Seq

##### siPadi2 ATAC-seq processing

ATAC-seq siCtrl and siPadi2 replicates were processed separately following the standard ENCODE pipeline for ATAC-seq samples, https://www.encodeproject.org/pipelines/ENCPL792NWO/. Adapters were detected and trimmed with cutadapt 1.9.1 (Martin, 2011) and aligned to mouse genome (GRCm38/mm10) using Bowtie2 (Langmead et al., 2009), with default parameters. After filtering mitochondrial DNA, reads properly paired were retained and multimapped reads, with MAPQ < 30, were removed using SAMtools (Li et al., 2009). PCR duplicates were removed using MarkDuplicates (Picard - latest version 1.126), http://broadinstitute.github.io/picard/.

ATAC-seq peaks were called using MACS2 https://github.com/taoliu/MACS/ (Zhang et al., 2008), with parameters -g mm -q 0.05–nomodel–shift −100–extsize 200 -B –broad. To get the consensus promoter/enhancer regions from each of the replicates we first selected the significant peaks -log10(p value) >1.30103. Using bedtools.2.17 mergeBed, we recovered the consensus ATAC peak region that included more than one replicate for siCtrl samples and siPadi2 samples. From where we recovered 76021 and 55772 union peaks regions for siCtrl and siPadi2, respectively. For visualization and further analyses, the different replicates were merged using Samtools and tracks were normalized using deeptools bamcoverage, normalized with RPKM.

##### H3K27ac ChIP-Seq Data

Raw reads were mapped to the mouse genome (GRCm38/mm10) with Bowtie 2.1.0 (Langmead et al., 2009). Uniquely mapped reads (17 and 19.5 million for proliferation and differentiation chromatin samples) were then processed with SAMTools for format conversion and removal of PCR duplicates (Li et al., 2009). Broad peaks corresponding to H3K27ac enrichment were called using MACS 2.0.10, with q-value cutoff at 5^∗^10-2 (Zhang et al., 2008). H3K27ac peaks were filtered against blacklisted genomic regions prone to artifacts (ENCODE Project Consortium, 2012) with BEDTools (Quinlan and Hall, 2010) and annotated to the nearest Transcription Start Site with PeakAnalyzer (Salmon-Divon et al., 2010).

#### Defined Promoter/Enhancer Regions

We recovered the regions around the transcription start site (TSS) from gene annotations, ENSEMBL83. The putative promoter regions were extracted as −3KB upstream the TSS and 100bp downstream the TSS, to recover possible promoter and enhancer regions. To get more reliable signal we intersected the siCtrl union peaks with peaks from H3K27ac ChIP-seq in Oli-neu cells in differentiation and proliferation conditions (see above), ending with 17281 siCtrl/promoter peaks overlapping with H3K27ac peaks. Only the union peaks (7087 peaks) from siCtrl/H3K27ac overlapping the defined promoter regions were retained, using Bedtools intersect (Quinlan and Hall, 2010). In order to recover possible enhancer and promoter regions that could be have been excluded from H3K27ac filtering or due to been more downstream of defined promoter regions, we included in the analysis a known mark of promoters/enhancers, H3K4me3 in OPC-like cells (GSE80089) (Lu et al., 2016). We recovered the peaks bed file of H3K4me3 control peaks, GSM2112628, (liftover mm9 to mm10) and with bedtools intersect, we recovered 10428 peaks corresponding to siCtrl/promoter/enhancer peaks with H3K4me3 peaks regions. The final dataset of peaks included the union of both promoter/enhancer peaks marked by H3K27ac and/or H3K4me3, with a total of 10897 peaks. The final region was extended 200bp up and downstream for further calculations.

For the differential accessibility analysis between conditions the merged replicates from both conditions were used with Pyicos (Althammer et al., 2011) on the final peaks dataset, with parameters pyicoenrich–tmm-norm and default parameters. Including the z-score associated p value and Benjamini & Hochberg (1995) corrected p value (adj p value or FDR) and the log2 fold change for candidates selection, with adjust p value < 0.05 significance threshold.

Data were visualized with IGV, http://www.broadinstitute.org/igv.

#### Gene Ontology Analysis

Gene ontology (GO) enrichment analysis for proteomics data were performed using STRING protein-protein interaction networks (Szklarczyk et al., 2017), and results adjusted for multiple testing using the Benjamini–Hochberg procedure (FDR).

For the GO analyses in ATAC-seq, the top 300 up genes from siCtrl selected peaks and top 300 genes form siPadi2 selected peaks, based on log2(RPKM siCtrl / RPKM siPadi2) were selected (adjusted p-val < 0.05). GO and pathway analysis was performed with the ClueGO (version 2.5.1) (Bindea et al., 2009) plug-in Cytoscape (version 3.5.1) (Smoot et al., 2011) with settings, GO Biological process (20.11.2017) and REACTOME pathways (20.11.2017), showing only pathways with p-val ≤ 0.05. Default settings were used and a minimum of 4 genes per cluster were used.

### Quantification and Statistical Analysis

Statistical analysis was performed in GraphPad Prism7. A parametric and non-paired t test was used to determine statistically significant differences between two groups in qPCR experiments with the exception of the rat primary cultures, where a one sample t test was performed. One-way ANOVA was used to determine differences between three groups in the behavioral analysis of *Padi2* fKO. Mann Whitney tests were performed to compare Ct and *Padi2* cKO behavior.

### Data and Software Availability

The accession number for all raw data has been deposited in GEO: GSE115929.

## Supplemental Information

Document S1. Figures S1–S3

Table S1. Citrullination Targets Detected in Oli-neu Cells in Both Proliferation and 2 Days Differentiation, Related to Figure 5 SILAC was performed in control and Padi2-overexpressing oli-neu cells. Light peptides belong to the control and heavy peptides to Padi2-overexpression cell lines.

Table S2. Padi2 Interactors Detected in Oli-neu Cells in Both Proliferation and 2 Days Differentiation, Related to Figure 6 SILAC was performed in control and biotin-tagged Padi2 overexpressing oli-neu cells. Light peptides belong to the control and heavy peptides to biotin-tagged Padi2-overexpression cell lines.

Table S3. Differential Accessibility on siCtrl ATAC-Seq Peaks Intersecting H3K4me3/H3k27ac Marked Promoters and Enhancers, Related to Figure 7

Table S4. Gene Ontology Analysis for the Citrullination Targets, PADI2 Interactors, and Differential Accessible Gene Promoter and Enhancer Regions, Related to Figures 5, 6, and 7

Table S5. List of Primers Used for qPCR and Genotyping, Related to Figures 1, 2, and 7

Document S2. Article plus Supplemental Information
