# Supplementary material for: PAD2-Mediated Citrullination Contributes to Efficient Oligodendrocyte Differentiation and Myelination
Source: Cell Rep. 2019 Apr 23;27(4):1090–1102.e10. doi: 10.1016/j.celrep.2019.03.108 (PMC6486480; doi:10.1016/j.celrep.2019.03.108)
Supplement: Document S1. Figures S1–S3 [file mmc1.pdf]

Cell Reports, Volume *27*

Supplemental Information

PAD2-Mediated Citrullination Contributes to Efficient Oligodendrocyte Differentiation and Myelination

Ana Mendanha Falcão, Mandy Meijer, Antonella Scaglione, Puneet Rinwa, Eneritz Agirre, Jialiang Liang, Sara C. Larsen, Abeer Heskol, Rebecca Frawley, Michael Klingener, Manuel Varas-Godoy, Alexandre A.S.F. Raposo, Patrik Ernfors, Diogo S. Castro, Michael L. Nielsen, Patrizia Casaccia, and Gonçalo Castelo-Branco

Falcao et al, 2019 - Supplementary Figure 1

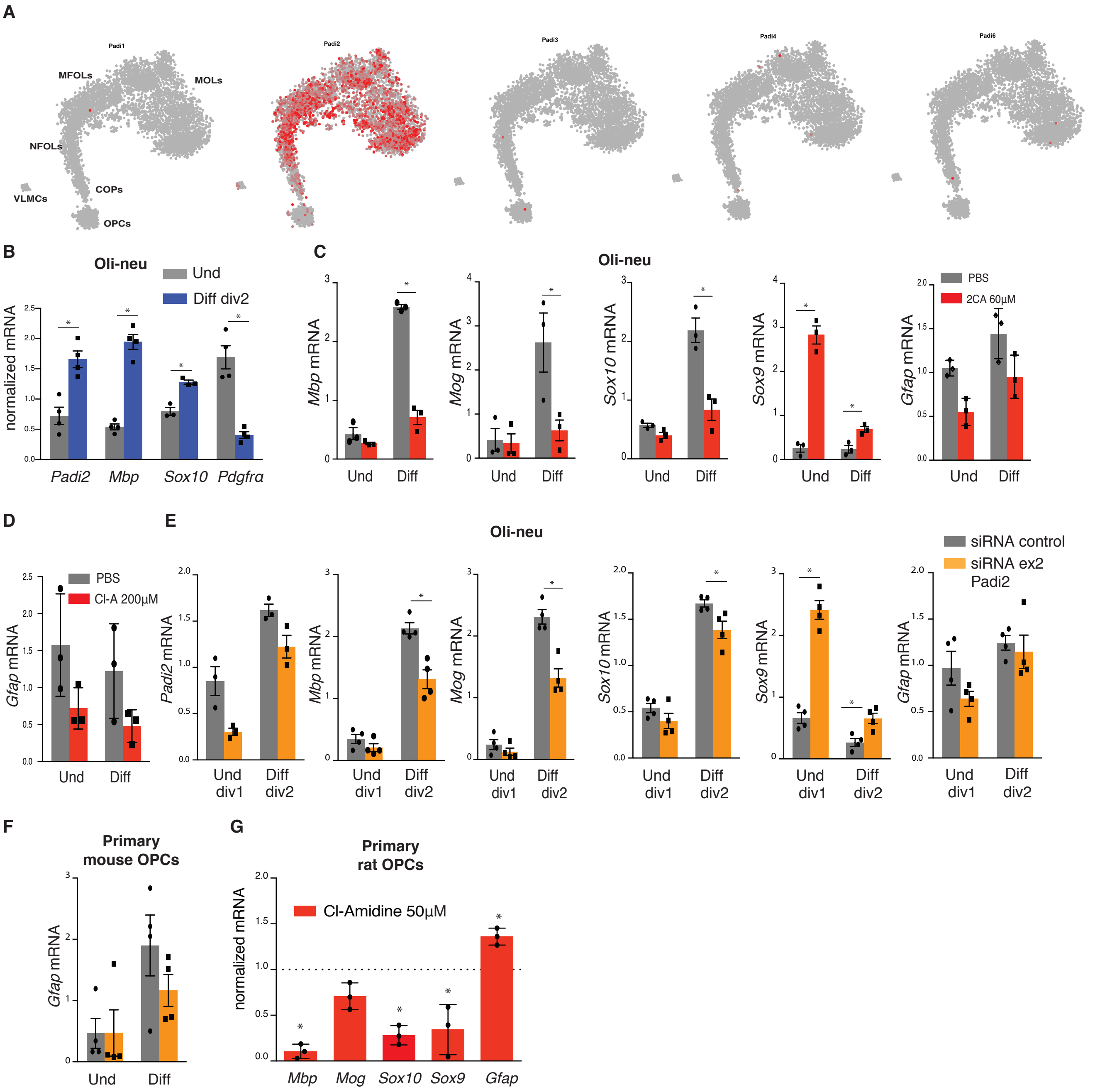

Supplementary Figure S1 (related to Figure 1 and 2). Inhibition of PADs and *Padi2* knockdown impairs Oli-neu differentiation.

(A) Gene expression of *Padi* family in the juvenile and adult oligodendrocyte lineage. Overlay in t-SNE from Marques et al., ref 15. Scale gray - low expression, red - high expression).

(B) Comparative gene expression analysis of undifferentiated (Und) and 2-days differentiated (Diff) Oli-neu cells. Means± SEM are shown, n=4, two-tailed t-test * p<0.05.

(C, D) Comparative gene expression analysis of undifferentiated (Und) and 2-days differentiated (Diff) Oli-neu cells treated either with PBS or with the PAD inhibitor 2CA (C) and Cl-Amidine (for Gfap) (D). Means ± SEM are shown, n=3, two-tailed t-test * p<0.05.

(E) Comparative gene expression analysis on undifferentiated (Und) and 1-day differentiated Oli-neu cells (Diff) transfected with either scramble siRNA or Padi2 siRNA targeting exon2. Cells were collected at day in vitro (div)1 or div2 upon transfection. Means ± SEM are shown, n=4, two-tailed t-test * p<0.05.

(F) Gfap mRNA transcription analysis on undifferentiated (Und) and 2-days differentiated primary OLs (Diff) transfected with either scrambled siRNA or Padi2 siRNA targeting exon2. Means ± SEM are shown, n=4 two tailed t-test *p<0.05.

(G) Gene expression analysis on proliferating rat primary OPCs treated with Cl-Amidine 50mM, dashed line represents control levels, normalized to 1. Means ± SEM are shown, n=3, one-sample t test *p<0.05.

Falcao et al, 2019 - Supplementary Figure 2

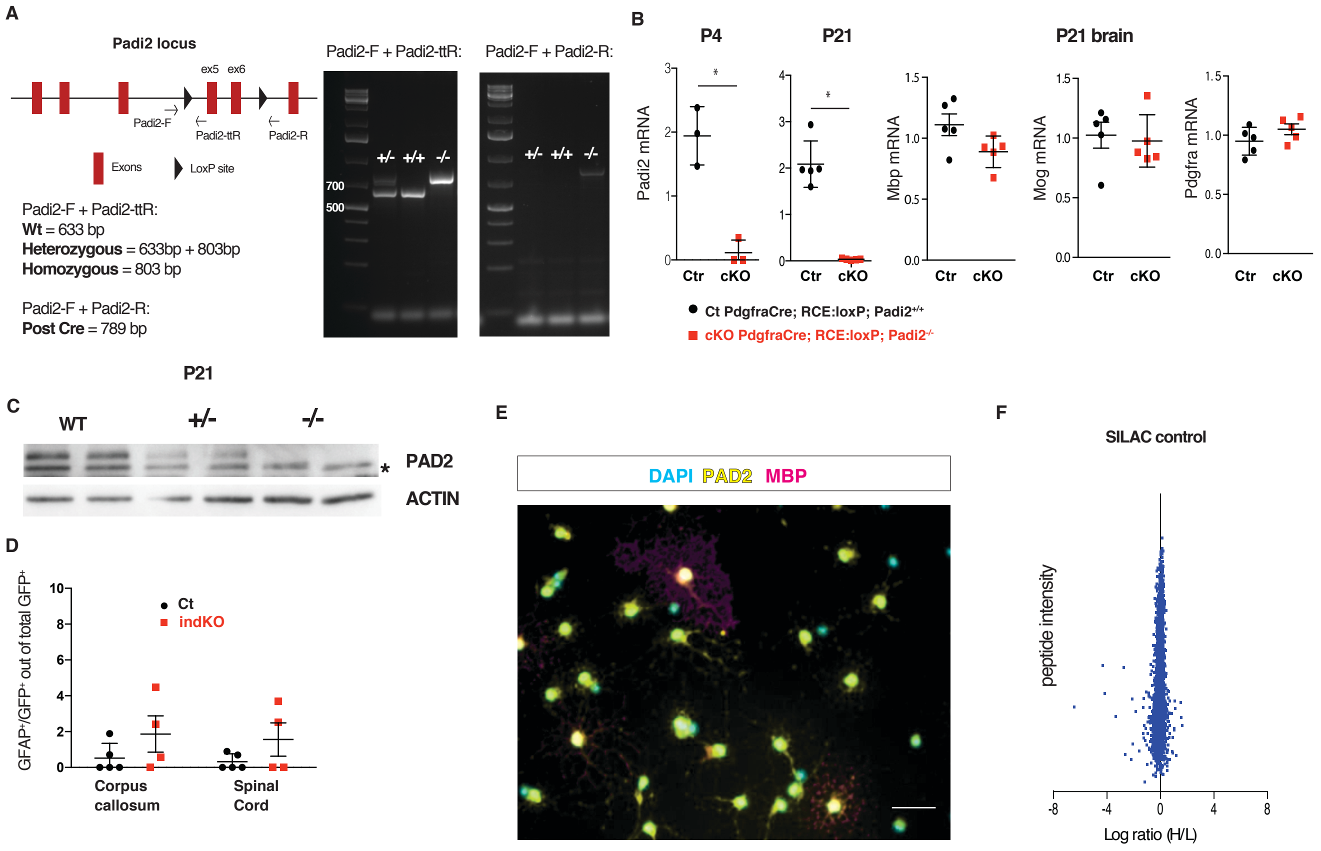

Supplementary Figure S2 (related to Figure 3, 4 and 5). Analysis of the PAD2 KO mice used in the study.
(A) Strategy used to genotype floxed Padi2 mice and to assess Cre/CreERT-mediated recombination. Primers Padi2-F + Padi2-ttR are designed amplify a non-recombined DNA sequence including floxed sequences in hetero and homozygous mice. Primers Padi2-F + Padi2-R detect a DNA sequence post Cre/CreERT mediated recombination. PCR product sizes for these PCRs are shown. Agarose gels depicting PCR products and the corresponding genotyping are shown. PCRs are performed for the ear and/or tail of mice that comprises both Pdgfra positive and negative cells allowing the observation of both floxed pre-cre and post-cre sequences.
(B) GFP+ cells were FACS sorted from P4 and P21 controls (Ctr, PdgfraCre; RCE-loxP; Padi2+/+) and Padi2 conditional knockouts (Padi2 cKO, PdgfraCre; RCE-loxP; Padi2-/-). Padi2, Mbp, Mog and Pdgfra mRNA expression levels were compared. Means ± SEM are shown, n=4 (P4) and n=5 (P21), two-tailed t test * p<0.05.
(C) Western blot for PAD2 in Wt and fPAD2 KO depicting the absence of PAD2 protein in the KO. * depicts a non-specific band.
(D) GFAP and GFP double positive cells were estimated out of total GFP+ cells both in corpus callosum and in spinal cord of PdgfraCreERT; RCE-loxP; Padi2+/+ and PdgfraCreERT; RCE-loxP; Padi2-/- mice. Means ± SEM are shown, n=5 and n=4, two-tailed t- test * p<0.05.
(E) Immunocytochemistry in 2-days mouse primary differentiated OLs for PAD2 (in yellow) and MBP (in magenta) depicting PADI2 in the nucleus and cytoplasm of MBP posititive and MBP negative cells. Scale bar = 20µm.
(F) Incorporation of isotopes in Oli-neu cells was highly efficient once mixing proteins of LIGHT and HEAVY labeled control cells, resulting in an average ratio of 1, or log ratio (H/L)=0.

Falcao et al, 2019 - Supplementary Figure 3

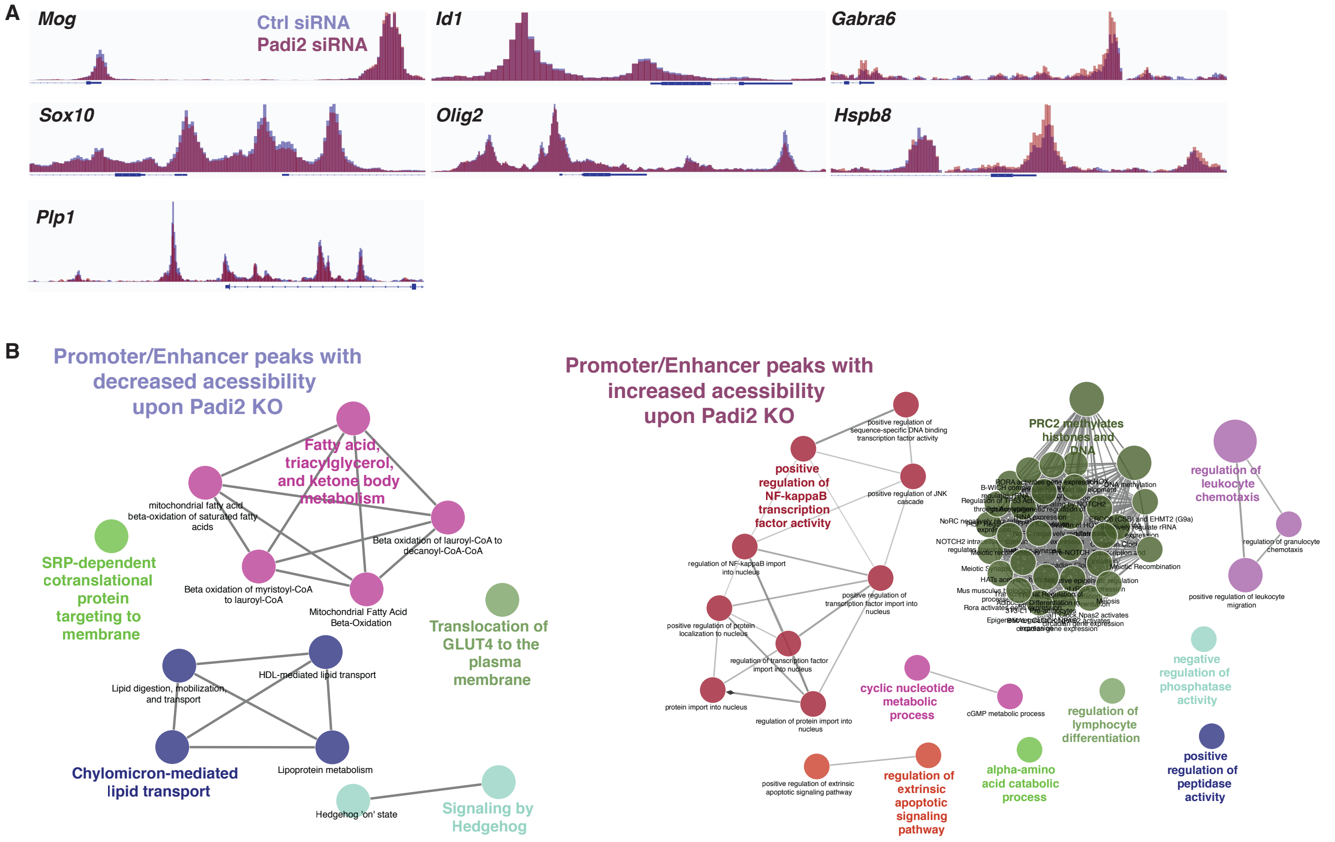

Supplementary Figure 3 (related to Figure 7). Padi2 knockdown exhibit altered chromatin accessibility

(A) IGV genome browser overlay views

depicting chromatin accessibility (assessed by ATAC-Seq) near transcription start sites, in Oli-neu transfected with Ctrl siRNAs (blue) and siRNA against Padi2(red) (n=3, samples pooled for visualization, same scale for ctrl siRNA and Padi2 siRNA).

(B) Gene ontology analysis of genes who present regulatory regions with increased or decreased chromatin accessibility upon Padi2 knockdown in Oli-neu cells.
